# The reference genome and transcriptome of the limestone langur, *Trachypithecus leucocephalus*, reveal expansion of genes related to alkali tolerance

**DOI:** 10.1186/s12915-021-00998-2

**Published:** 2021-04-08

**Authors:** Tengcheng Que, Huifeng Wang, Weifei Yang, Jianbao Wu, Chenyang Hou, Surui Pei, Qunying Wu, Liu Ming Li, Shilu Wei, Xing Xie, Hongli Huang, Panyu Chen, Yiming Huang, Aiqiong Wu, Meihong He, Dengpan Nong, Xiao Wei, Junyi Wu, Ru Nong, Ning Huang, Qingniao Zhou, Yaowang Lin, Tingxi Lu, Yongjie Wei, Shousheng Li, Jianglong Yao, Yanli Zhong, Huayong Qin, Luohao Tan, Yingjiao Li, Weidong Li, Tao Liu, Sanyang Liu, Yongyi Yu, Hong Qiu, Yonghua Jiang, Youcheng Li, Zhijin Liu, Cheng Ming Huang, Yanling Hu

**Affiliations:** 1 Terrestrial Wildlife Rescue and Epidemic Diseases Surveillance Center of Guangxi, Nanning, Guangxi 530003 China; 2 Department of Biochemistry and Molecular Biology, School of Pre-Clinical Medicine, Guangxi Medical University, Nanning, Guangxi 530021 China; 3 Annoroad Gene Technology, Beijing, 100176 China; 4 Guangxi Chongzuo white headed langur national nature reserve, Chongzuo, Guangxi 532200 China; 5 School of Information and Management, Guangxi Medical University, Nanning, Guangxi 530021 China; 6 Guangxi Reproductive Medical Research Center, First Affiliated Hospital of Guangxi Medical University, Nanning, Guangxi 530021 China; 7 Life Sciences Institute, Guangxi Medical University, Nanning, Guangxi 530021 China; 8 Center for Genomic and Personalized Medicine, Guangxi Medical University, Nanning, Guangxi 530021 China; 9 Nanning Animal Zoo, Nanning, Guangxi 530021 China; 10 College of Life Sciences, Capital Normal University, Beijing, 100048 China

**Keywords:** *Trachypithecus leucocephalus*, Genome assembly, Limestone karsts, Alkali tolerance, Mineral ion binding

## Abstract

**Background:**

*Trachypithecus leucocephalus*, the white-headed langur, is a critically endangered primate that is endemic to the karst mountains in the southern Guangxi province of China. Studying the genomic and transcriptomic mechanisms underlying its local adaptation could help explain its persistence within a highly specialized ecological niche.

**Results:**

In this study, we used PacBio sequencing and optical assembly and Hi-C analysis to create a high-quality de novo assembly of the *T. leucocephalus* genome. Annotation and functional enrichment revealed many genes involved in metabolism, transport, and homeostasis, and almost all of the positively selected genes were related to mineral ion binding. The transcriptomes of 12 tissues from three *T. leucocephalus* individuals showed that the great majority of genes involved in mineral absorption and calcium signaling were expressed, and their gene families were significantly expanded. For example, *FTH1* primarily functions in iron storage and had 20 expanded copies.

**Conclusions:**

These results increase our understanding of the evolution of alkali tolerance and other traits necessary for the persistence of *T. leucocephalus* within an ecologically unique limestone karst environment.

## Background

Natural terrestrial islands are useful for exploring the factors influencing biological evolution, including patterns of colonization, adaptation, and diversification [1]. In some cases, traits have evolved to allow species to survive in extreme environments. High-quality genome sequencing is an effective method for studying the mechanisms underlying local adaptation. For example, the genome of the sooty mangabey (*Cercocebus atys*) contains a C-terminal frameshift in the *TLR4* gene, and a mutation in exons 3 and 4 of the ICAM2 protein might contribute to natural AIDS resistance [2]. In the Egyptian rosette bat (*Rousettus aegyptiacus*), an expanded and diversified KLRC/KLRD family of natural killer cell receptors, type I interferons, and MHC class I genes strongly contribute to antiviral defense [3]. The reference genome and transcriptome of the Indian cobra allow for the comprehensive identification of venom toxins [4]. These studies illustrate that knowledge of the structure of animal genomes is highly informative for physiological ecology, metabolism evolution, and local adaptation.

Caused by the weathering of limestone over time, karst mountains typically contain alkaline soil, abundant cliffs, and extremely dry conditions on porous limestone bedrock. Karst habitats cover extensive areas and are often ecologically fragile, with rich plant diversity and large numbers of endemic species [5, 6]. With the erosion of limestone, the concentrations of elements such as K^+^, Na^+^, Ca^2+^, and Mg^2+^ tend to increase in nearby soil and water. This geochemistry can affect local organisms. For example, higher mineral concentrations are found in the leaves of deciduous trees in karst forests [7]. It is therefore meaningful to explore whether species living on limestone mountains exhibit increased alkali tolerance.

One group of primates known to live in karst mountains are the limestone langurs. Endemic to Southeast Asia, the colobine genus *Trachypithecus* contains seven species of limestone langurs, namely *T. leucocephalus*, *T. francoisi*, *T. delacouri*, *T. poliocephalus*, *T. laotum*, *T. ebenus*, and *T. hatinhensis*. Although limestone langurs are adapted to a highly alkaline environment, high-quality genome-wide sequencing has only been performed for *T. francoisi*. Comparative genomics of *T. francoisi* and re-sequencing of other Asian *Trachypithecus* species revealed an adaptation to the naturally high levels of calcium in the water and plant resources in karst habitats [8]. In addition to the adaptation to high calcium levels, adaptation to highly alkaline environments, including high concentrations of minerals and metal ions, is required for limestone langurs to live in their native habitat.

*Trachypithecus leucocephalus* (*T. leucocephalus*), the white-headed langur, is restricted to a 200-km^2^ triangular area bounded by the Zuojiang River, the Mingjiang River, and the Sifangling Mountains in Guangxi province, China [9]. As it is endemic to limestone karsts, this species possesses unique adaptations that make it a particularly interesting model for studying the mechanisms underlying the limestone karst speciation. Several studies have assessed the genetics of *T. leucocephalus*. For example, Wang et al. [10] applied sequencing of the mitochondrial control region to 390 fecal samples from 40 social groups across the distribution of *T. leucocephalus*. They found remarkably low genetic diversity in this species. After subsequent genotyping of 15 polymorphic autosomal microsatellite loci and the mitochondrial hypervariable region I, they found that intragroup genetic correlations were stronger than intergroup correlations [11]. However, high-quality genomic studies on the evolutionary history of *T. leucocephalus* and their unusual adaptations to limestone karsts mountains are limited. To examine the potential role of genetic markers in the local adaptation, and ultimately conservation, of *T. leucocephalus*, we performed high-quality whole-genome sequencing using PacBio and optical assembly with chromosome conformation capture. This generated 2612 consensus contigs (~ 2.85 Gb) with a contig N50 of ~ 5.6 Mb. Simultaneously, we sequenced the transcriptomes of 12 tissues from three *T. leucocephalus* to further assess the genome function. In this study, we aimed to uncover the genetic changes underlying the specific adaptations of *T. leucocephalus* to the unique environment posed by limestone mountains.

## Results

### Sequencing and assembly of the *T. leucocephalus* genome

To obtain a high-quality *T. leucocephalus* genome, we first collected Illumina paired-end reads (150 bp) and described their heterozygosity and repeat characteristics. We obtained nearly 170 Gb of paired-end reads in total (Table S1). The observed level of heterozygosity was high (~ 0.35%), and the repeat content was low (~ 22%). The estimated genome size was 2.8 Gb, based on the *k*-mer analysis (*k* = 21, Table S2).

To further improve the genome reference assembly, we collected ~ 180 Gb subreads (N50 = 11,911 bp) generated from the PacBio Sequel platform with 60× coverage. The library insert was 20 kb, with 29 cells, and the mean length was 8294 bp. The genome from SMARTdenovo maintained high performance (Table S3). We used Canu (v1.8) [12] for self-correction and Arrow (SmartLink 5.0) and Pilon (v1.22) [13] for Illumina reads (~ 60×) to generate 2612 consensus contigs (~ 2.85 Gb), with a contig N50 of ~ 5.6 Mb (Table 1). To evaluate genome quality, we mapped all Illumina DNA reads back to the genome with a 99.74% mapping rate and a 99.65% coverage rate (Table S4). Using the *T. leucocephalus* blood sample, we mapped the 114,177 ESTs assembled using Trinity (v2.8.4) [14] from Illumina reads back to the assembled genome and found that 94% of the ESTs could be mapped to one contig with 90% coverage (Table S5). We evaluated the quality of the assembly using 4104 Benchmarking Universal Single-Copy Ortholog (BUSCO) genes from the mammalia_odb9 database. Among these genes, 3872 (~ 94.3%) were annotated entirely and 114 were not annotated (Table 1).

**Table 1:** Statistics of the assembled *T. leucocephalus* genome

Type	*T. leucocephalus*
Genome size (Mb)	2848.3
Contig N50 (kb)	5600
Scaffold N50 (kb)	–
Number of contigs	2612
Number of scaffolds	–
Maximum length of sequence contig (kb)	40,301.6
Maximum length of sequence scaffold (kb)	–
GC content (%)	41.4
Illumina mapping rate (%)	99.7
Transposable element (TE) proportion	52.8
Annotated protein-coding genes	20,925
Complete BUSCOs	94.3
Fragmented BUSCOs	2.9
Missing BUSCOs	2.8

*Note*: *BUSCOs* or Benchmarking Universal Single-Copy Ortholog genes constituted 4104 core proteins from the mammalia_odb9 database

To further anchor and orient the contigs into super-scaffolds, we constructed a Hi-C library from the *T. leucocephalus* blood sample. We generated ~ 245 Gb of data (~ 87× coverage; Table S6). Considering that the Hi-C interaction intensity decreased as the physical distance between contigs in a chromosome increased, we clustered and oriented 888 contigs with lengths of 2.6 Gb (~ 92%) into 22 super-scaffolds (N50 = 130 Mb) using a hierarchical clustering strategy from LACHESIS (Table S6). The distinct anti-diagonal pattern observed in Hi-C interaction matrices may indicate that the super-scaffolds were correctly anchored and oriented.

### Gene structure and annotation

**Fig. 1:**
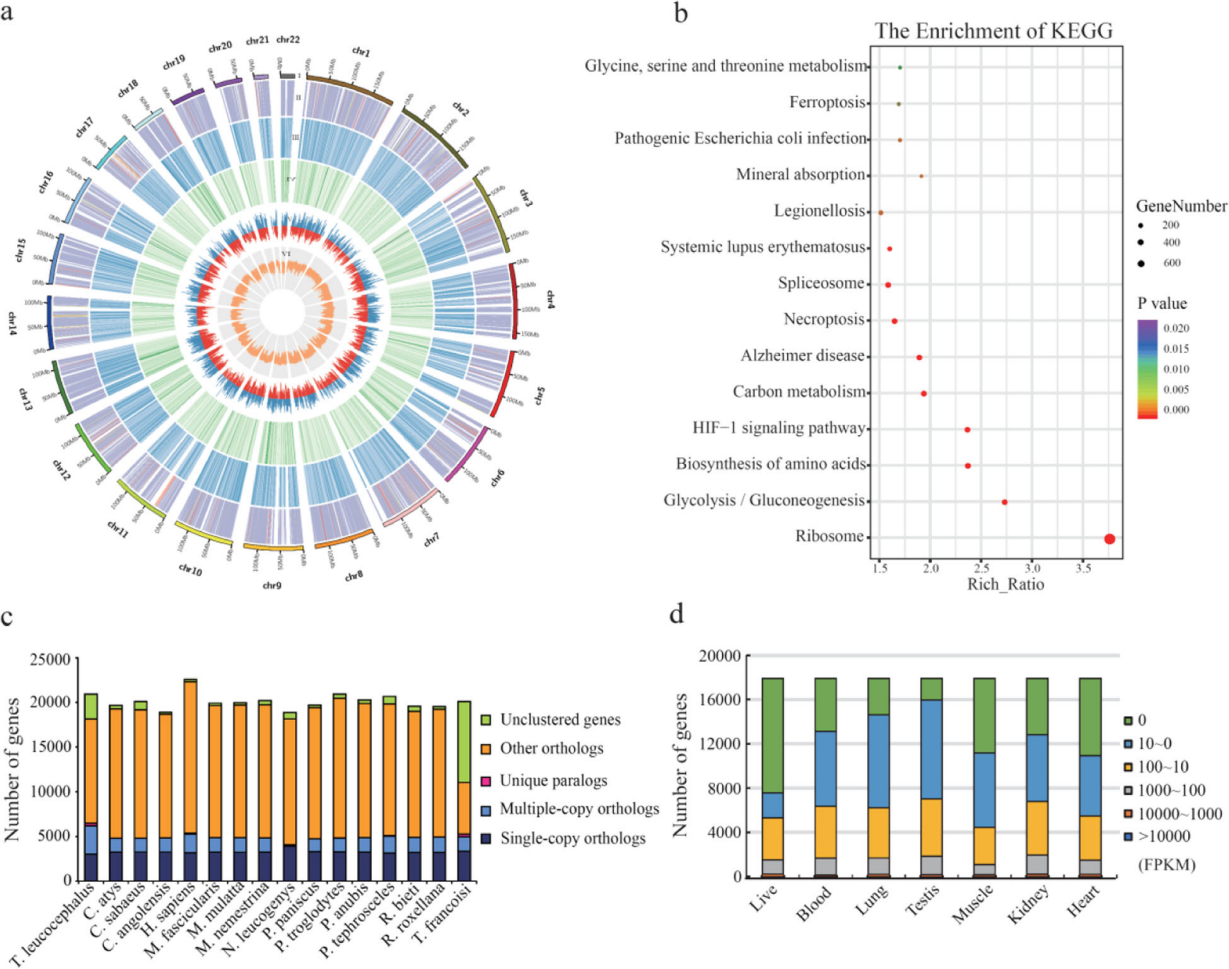
Genomic landscape of *T. leucocephalus*. **a** Circle diagram depicting the *T. leucocephalus* genome, divided into five categories, including noncoding RNA, gene abundance, transcriptome, GC content, and repeats. Starting at the outside of this circle, the divisions are as follows. I: Chromosome information. II: Noncoding RNA information in the 10 k statistic unit, including the number and distribution of rRNA, tRNA, and other types of noncoding RNA. III: Repeat information in the 10 k statistic unit. Darker colors indicate more highly significant values. IV: The abundance of genes in the 100 k statistic units. Darker colors indicate more highly significant values. V: Blood expression levels with log_2_ (FPKM) are shown in red (negative) and blue (positive). VI: GC content information in the 10 k statistic units. **b** KEGG pathway enrichment with over 200 genes. **c** Orthologs of gene families in 16 primates. **d** Whole-genome transcription information of seven tissues from *T. leucocephalus*

We combined homology and de novo assembly with the RNA-Seq method to elucidate the structure and final annotation of protein-coding genes. We predicted 20,945 protein-coding genes with an average transcript length of 15,390 bp. The protein-coding gene number in the *T. leucocephalus* genome was similar to that of *Rhinopithecus roxellana*, and chromosome sequences contained over 18,925 genes (~ 90% of the total). Nearly 1.69 genes were contained in each of 11,149 blocks spanning 100 kb. Genes were unevenly distributed across the genome and were more abundant at the ends of each chromosome (Fig. 1a). Chromosome 13 contained the greatest density of genes, and 1317 of them encode proteins. To identify proteins and their functions, and to annotate each gene, we aligned protein-coding sequences to sequences from various databases (Nr, KEGG, PFAM, GO, Swiss-Prot, and EggNOG). We found that, of the 20,945 total protein-coding genes, 59.7%, 96%, 75.5%, and 82.9% had homologs in the KEGG, Nr, Pfam, and EggNOG databases, respectively. We also observed that 20,131 genes (96.2%) encoded proteins that were homologous to proteins in at least one protein database (Table S7), and 338 pathways in the KEGG database were detected (Fig. 1b). A total of 736 transfer RNAs (tRNAs), 11,113 microRNAs (miRNAs), 2790 small nuclear RNAs (snRNAs; including C/D box, H/ACA box, and splicing), and 736 ribosomal RNAs (rRNAs; including 18S, 28S, 5.8S, and 5S) were identified in the *T. leucocephalus* genome (Table S8). The classification of orthologs from 16 primates showed that *T. leucocephalus* had more multiple-copy orthologs (Fig. 1c).

To identify known repetitive elements in the assembled genome, we employed the Repeat Modeler and TRF software for de novo repeat class prediction using RepeatMasker (open-4.0.6) and RepeatProteinMask (v.4.0.6) [15], aligned with the Repbase database. We merged and annotated all of the identified repeats through Repbase classification. Our analysis revealed that 1515 Mb (53.2% of all sequences) were repeats. RepeatMasker identified 51.04% of sequences as repeats. These values are slightly higher than those of *C. atys* (48.43%), *Rhinopithecus bieti* (47.6%), and *R. roxellana* (49.93%). The LINE type TE classification retained nearly all of the classes (26.39%, ~ 629 Mb; Table S9).

### Comparative genomic analysis

To distinguish gene families, we performed an all-vs-all comparison between *T. leucocephalus*, *T. francoisi*, and 14 closely related species, namely *Nomascus leucogenys*, *Homo sapiens*, *Pan troglodytes*, *Pan paniscus*, *Piliocolobus tephrosceles*, *R. roxellana*, *R. bieti*, *Chlorocebus sabaeus*, *Papio anubis*, *Macaca nemestrina*, *Macaca mulatta*, *Macaca fascicularis*, *C. atys*, and *Colobus angolensis* (Table S10). We detected 2772 single-copy orthologous genes in the *T. leucocephalus* genome (Table S11) and identified 22,458 gene families among the 16 closely related species. Of these, 302 gene families were private to *T. leucocephalus*. In addition, whole-genome transcription scanning of 12 tissues from three *T. leucocephalus* indicated there were many genes being expressed in the lungs and testes (Fig. 1d). Enrichment analysis of 292 genes present only in *T. leucocephalus* showed calcium signaling pathways to be in the top 10 enriched pathways (Table S12). According to GO analysis, 66 of these 292 unique genes were related to alkaline ion processing, such as sodium ion transmembrane transport, potassium ion transport, calcium signaling, metal ion binding, and metallocarboxypeptidase activity (Table S13). Most of these unique genes were related to metal or calcium ion binding.

**Fig. 2:**
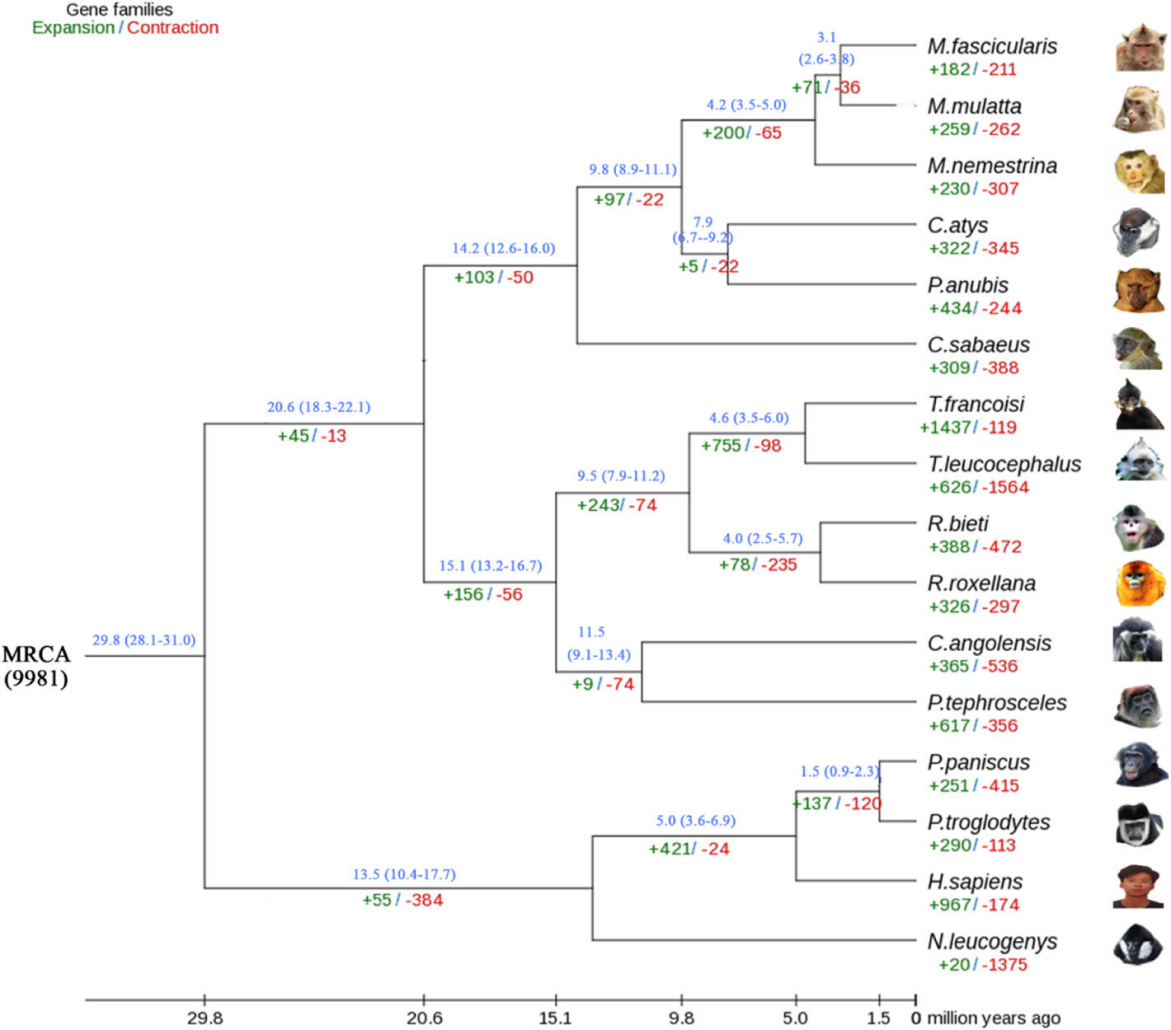
Phylogenetic tree of *T. leucocephalus* and 15 other primate species. A maximum likelihood tree based on 5345 orthologous proteins was generated and used to infer the expansion and contraction of 9906 gene families. The numbers of expanded and contracted gene families are shown in green and red, respectively. The estimates of divergence time are shown in blue. The number by the most recent common ancestor (MRCA; at the root of the tree) indicates the bootstrap evidence for partition based on 1000 bootstrap replicates

In addition to comparing genome structure and function among *T. leucocephalus*, *T. francoisi*, and 14 other primate species, we assessed their evolutionary relationships by inferring homologous protein groups. We detected 5345 single-copy orthologous genes and aligned their protein sequences to construct a phylogenetic tree through maximum likelihood estimation (Fig. 2). We found that species in the *Trachypithecus* and *Rhinopithecus* genera were relatively closely related, with a divergence time close to 9.5 million years ago (Mya). *T. leucocephalus* and *T. francoisi* appear to have differentiated from each other approximately 4.6 Mya.

We identified 1078 expanded gene families (327 significant at the *P* < 0.05 level; Table S14) and 428 contracted gene families (137 significant at the *P* < 0.05 level; Table S15). Annotation and functional enrichment of the expanded gene families revealed that many genes involved in metal ion binding, mineral ion transport and homeostasis, calcium signaling, and calcium ion binding were significantly enriched. The numbers of expanded gene families involved in metal or calcium ion binding were particularly notable. For example, the molecular function branch of the GO database annotated *PCDHA6* and *PCDHGA9*, with 11 and 8 copies, respectively, as being involved in calcium ion binding. In the *T. leucocephalus* genome, *PCDHA6* and PCDHGA9 were annotated as scaffolds (Fig. S1A, B). In the *T. leucocephalus* transcriptome, almost all *PCDHA6* and *PCDHGA9* members were expressed in at least one kidney (Fig. S1C, D).

After aligning sequences in PRANK [16], we used branch-site likelihood ratio tests to uncover 207 genes that were more strongly positively selected in the *T. leucocephalus* genome compared to the genomes of the other primates we studied (Table S16). Of these 207 positively selected genes (PSGs), 66 were annotated to mineral ion binding and transport, such as sodium ion transport, metal ion binding, ion homeostasis, salt ion binding, and calcium ion binding. Almost all of the PSGs were at least indirectly related to mineral ion binding. For example, the PSG *F341I* was found within *SCN8A*, which encodes a member of the sodium channel α-subunit gene family and is therefore important for sodium ion transport [17]. The residue analysis and 3D model showed that SCN8A residue was located in this protein (Fig. S2A–C). The Ile residue in the mutation can chelate metal ions, and its location should not affect the structure of this protein. This evolution could strengthen both the ability to bind ions and tolerance to highly alkaline conditions.

### Functional evolution of the mineral absorption pathway

**Fig. 3:**
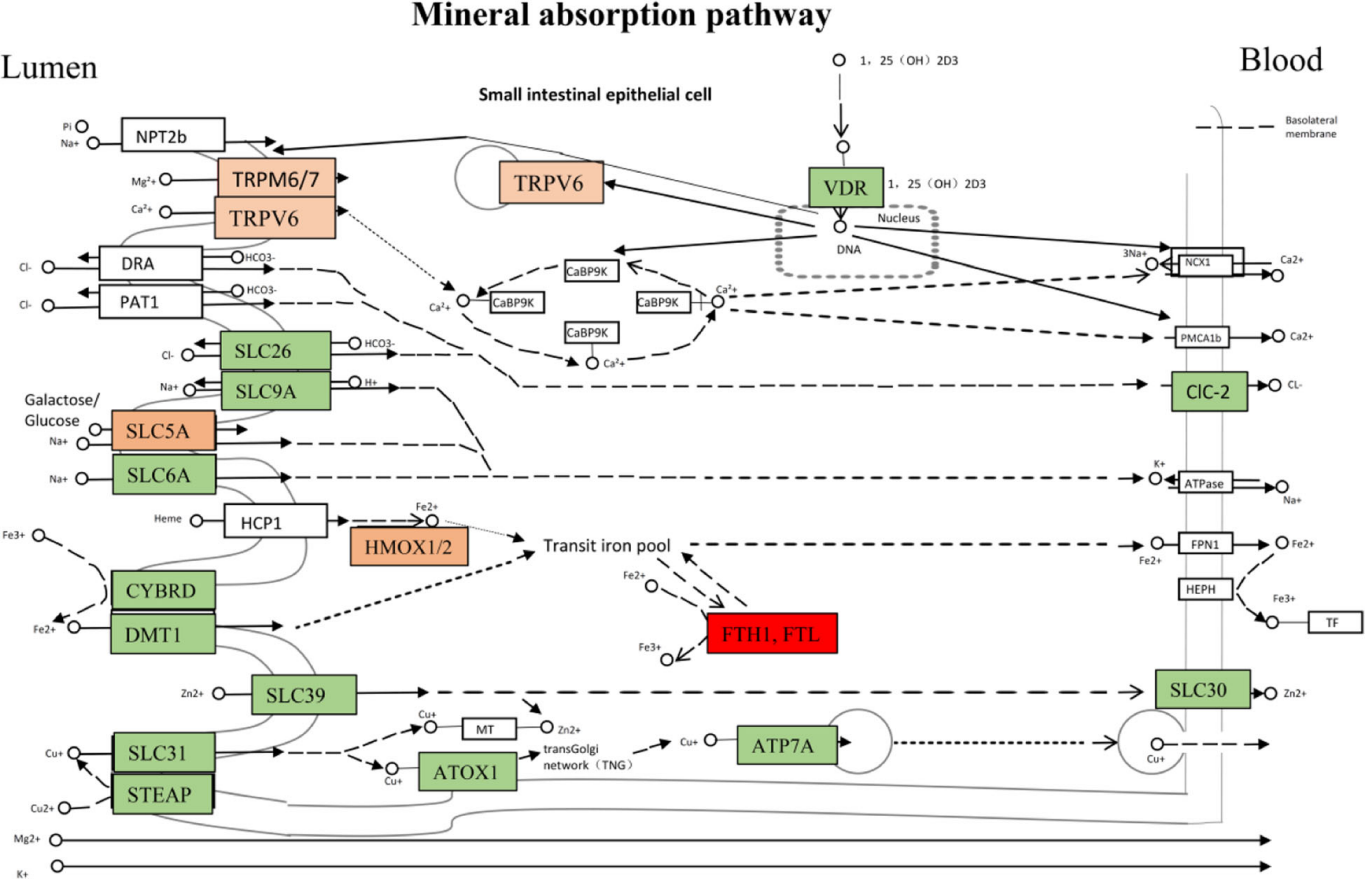
Schematic overview of the mineral absorption pathway in *T. leucocephalus*. The expression of genes in the mineral absorption pathway is indicated with different colors. Red denotes significant gene family expansion, and orange denotes nonsignificant gene family expansion. Green denotes expression in the signal of 12 transcriptome samples. White denotes no expression in the signal of 12 transcriptome samples

The ability of the body to effectively control the circulating levels of mineral ions, such as Ca^2+^, Mg^2+^, and Zn^2+^, is essential for development and health. In the genome of *T. leucocephalus*, most genes in the “mineral absorption” KEGG pathway were expressed (Fig. 3, Table S17). Within this pathway, eight gene families (*FTH1*, *FTL*, *TRPM6*, *HMOX2*, *SLC26*, *SLC30*, *SLC5A*, and *CLCN2*) are expanded in the *T. leucocephalus* genome, and the expansion of *FTH1* and *FTL* was statistically significant (*P* < 0.05, Fisher’s exact test). There are 20 copies of *FTH1* in the *T. leucocephalus* genome (*P* < 0.05, Fisher’s exact test). We constructed a phylogenetic tree based on 48 amino acid sequences from 15 primates and found that 15 copies represented monophyletic expansions and tightly clustered together, whereas five copies were scattered among several clusters belonging to various species (Fig. S3A). The functional importance of *FTH1* genes was further demonstrated through expression analysis of 12 transcriptomes from two *T. leucocephalus* individuals, and all 20 of the expanded copies of *FTH1* were expressed. Nine copies were found in the transcriptomes of all of the tissues (Fig. S3B, Table S18). *FTH1* encodes a heavy ferritin subunit of an intracellular iron storage protein in both prokaryotes and eukaryotes. A primary function of ferritin is the storage of iron in a soluble and nontoxic state [18, 19]. Induction of ferritin expression through exposure to 3H-1,2-dithiole-3-thione may be a novel therapy to treat valvular mineralization [20]. These observations suggest that these expanded heavy ferritin chain gene copies may enable *T. leucocephalus* to adapt to its highly specialized environment.

The transcriptome showed that almost all of the genes in the mineral absorption pathways (*TRPM6*, *ATOX1*, *HMOX1*, *HMOX2*, *SLC30*, and *VDR*) were expressed in all of the tissues, especially the liver. Transient receptor potential cation channel subfamily M member 6 (*TRPM6*) encodes a protein containing an ion channel domain and a protein kinase domain. This gene is important for Mg^2+^ uptake in vertebrate cells and magnesium homeostasis [21]. Schmitz et al. [22] showed that *TRPM6* plays an essential role in epithelial Mg^2+^ transport and in active magnesium absorption in the gut and kidney. Four copies of *TRPM6* and *ATOX1* were detected in the genome of *T. leucocephalus*. We compared five nonhuman primates—(i) *T. leucocephalus* from their natural habitat; (ii, iii) *M. mulatta* from a limestone karst [MRH] and caged [MCH]; (iv) *Hylobates*, caged; and (v) *Mandrillus sphinx*, caged—and found that *T. leucocephalus* had a significantly higher blood Mg^2+^ concentration (2.658 ± 0.165 mmol/L, *n* = 13) than the other four primates (MRH, 0.897 ± 0.028 mmol/L, *n* = 7; MCH, 0.976 ± 0.109 mmol/L, *n* = 10; gibbon, 1.046 ± 0.054 mmol/L, *n* = 14; *M. sphinx*, 1.152 ± 0.200 mmol/L, *n* = 6; all *P* < 0.0001, Student’s *t* test). There was no difference in Mg^2+^ levels between MRH and MCH (Fig. S4A). The blood K^+^, Na^+^, and Ca^2+^ concentrations of *T. leucocephalus* were also significantly higher than those of the other four primates (Fig. S4B–D). These results indicate that *T. leucocephalus* has adapted to high levels of alkaline ions.

### Functional evolution related to Ca^2+^ signaling

**Fig. 4:**
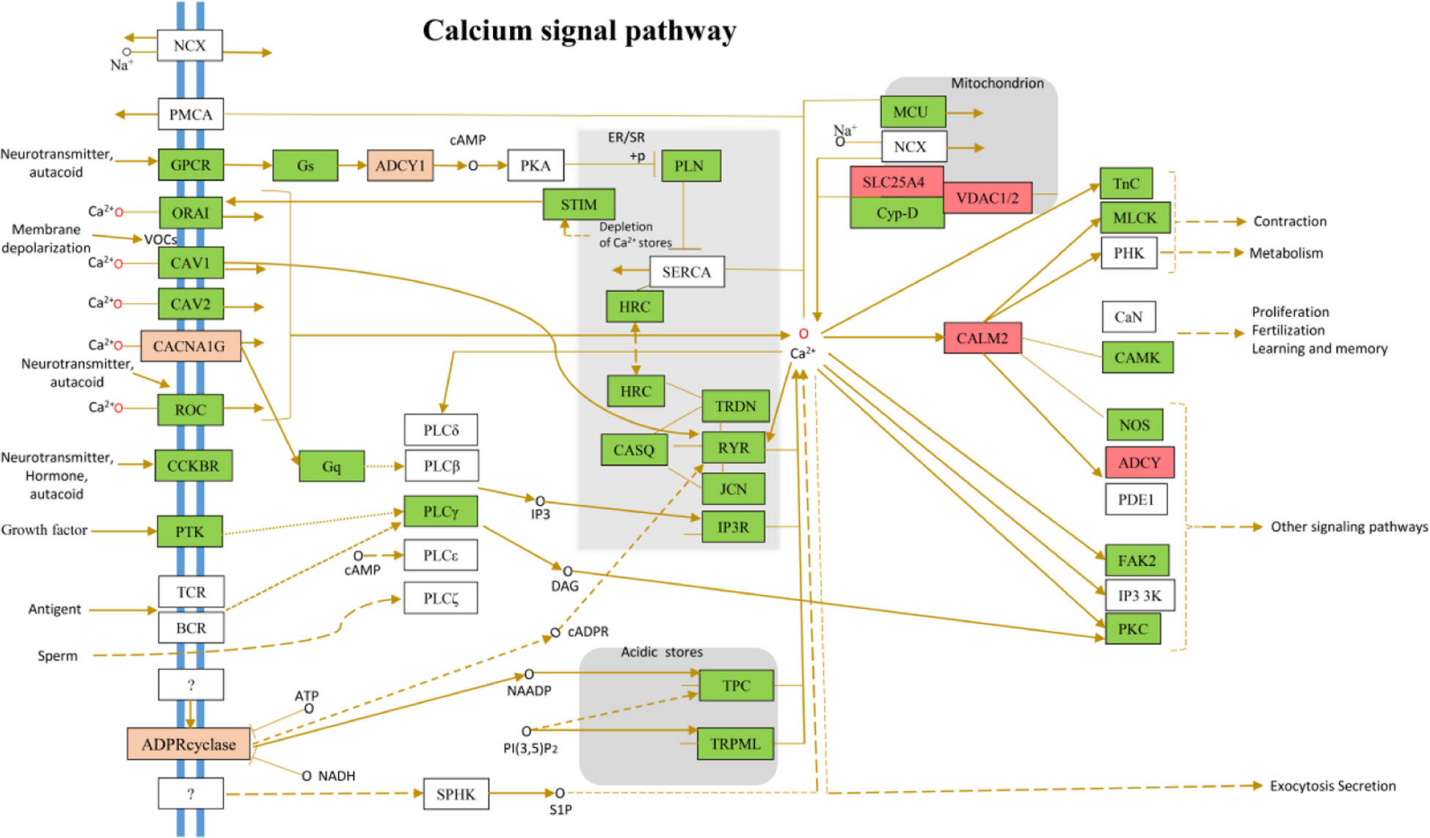
The calcium (or Ca2+) signaling pathway in *T. leucocephalus*. The expression of genes in the calcium signaling pathway is indicated with different colors. Red denotes significant gene family expansion, and orange denotes nonsignificant gene family expansion. Green denotes expression in the signal of 12 transcriptome samples. White denotes no expression in the signal of 12 transcriptome samples

Cells take up external Ca^2+^ by activating various entry channels that have widely different properties [23]. The influx of Ca^2+^ from the environment or release from internal stores causes a rapid and dramatic increase in the cytoplasmic calcium concentration, which plays an important role in Ca^2+^ signal transduction. We found that five significantly expanded families (*VDAC1*, *VDAC2*, *ADCY*, *CALM2*, and *SLC25A5*) and three PSGs (*ADCY1*, *CACNA1G*, and *CCKBR*; false discovery rate-adjusted *P* < 0.05, Fisher’s exact test) were enriched in Ca^2+^ signaling (Fig. 4).

The mitochondria are involved in necrotic cell death under Ca^2+^ overload, which leads to mitochondrial swelling and rupture. Voltage-dependent anion channels (VDACs) are mitochondrial porins that transport Ca^2+^ and other metabolites through the outer mitochondrial membrane (OMM). Through assessing the activity of cardiac mitochondria, an indicator of mitochondrial susceptibility to Ca^2+^-induced swelling, a comparison between wild-type and VDAC1-null mice indicated that the mitochondria lacking VDAC1 are more susceptible to swelling [24]. The VDAC1 mutant was deficient in monoubiquitination, which could accelerate apoptosis via the mitochondrial calcium uniporter channel [25]. Shimizu et al. [26] also verified the vital role of VDAC2 in regulating mitochondrial Ca^2+^ uptake for cardiac rhythmicity. In this study, *VDAC1* and *VDAC2* genes were expanded to six copies in the *T. leucocephalus* genome, but only one copy was found in all other primates studied. Four *VDAC* copies were expressed in all seven tissues assayed (Fig. S5A). Several amino acid changes occurred, leading to increased diversity of both VDAC1 and VDAC2 gene families in *T. leucocephalus* compared to the other 14 primates (Fig. S5B, C). Calmodulin can sense local changes in Ca^2+^ concentration across cell membranes and relay the information to numerous interaction partners. Genes encoding for CALM2, a member of the calmodulin family, are constitutively expressed and related to the modulation of the voltage-gated calcium Cav2.1 channel, which plays an essential role in the regulation of calcium homeostasis [27, 28]. Contrary to *VDAC1* and *VDAC2*, the ryanodine receptor (*RyR*) gene family was contracted in the *T. leucocephalus* genome. At higher Ca^2+^ concentrations, *RyR* could be activated to release Ca^2+^ from the endoplasmic reticulum (ER) [29], resulting in a reduction in ER Ca^2+^ levels [30, 31]. However, Ca^2+^ concentrations in the serum of *T. leucocephalus* were significantly higher than in other primates (Fig. S4D). The contraction of the *RyR* family can balance Ca^2+^ homeostasis in vivo.

The OMM is permeable to molecules, allowing for rapid transmembrane diffusion of Ca^2+^ [23]. Outer mitochondrial membrane permeability results primarily from the abundant expression of *VDAC* [32]. The mitochondrial Ca^2+^ uniporter (MCU) is a highly Ca^2+^-selective channel in the inner mitochondrial membrane. The mitochondria can take up large quantities of Ca^2+^ through the MCU, resulting in transient and sustained elevations of mitochondrial Ca^2+^ levels. Downregulation of MCU-related genes results in reduced mitochondrial and increased cytosolic Ca^2+^ levels [33]. In the *T. leucocephalus* genome, the MCU family is contracted, which could contribute to the regulation of Ca^2+^ homeostasis in the mitochondria.

## Discussion

Karst environments have topography characterized by the weathering of carbonate rocks, primarily limestone. Dissolution of limestone results in highly alkaline soil and water that are difficult for many plant and animal species to tolerate [34]. However, some species, such as *T.* leucocephalus, have evolved to withstand these harsh conditions. In this study, we applied PacBio sequencing and optimal assembly with Hi-C to obtain a high-quality *T. leucocephalus* genome, which provides novel phylogenetic and functional insights into primate evolution and elucidates the nature of genetic adaptations to karst mountain environments.

The mRNA expression profiles and blood mineral concentrations of caged *T. leucocephalus* showed that levels of Na^+^, K^+^, Mg^2+^, and Ca^2+^ were significantly higher in this species than in the other caged primates studied. Many minerals (such as iron) are essential for physiological processes. However, high concentrations of alkaline ions could cause alkalosis. KEGG pathway enrichment analysis showed that mineral absorption was an attribute of the most highly enriched pathways in *T. leucocephalus*, with more than 200 genes. Given high mineral concentrations, many of the unique and significantly expanded gene families in the *T. leucocephalus* genome encode ion binding proteins and peptides, which may reduce the mineral ion levels in vivo.

The Ca^2+^ binding sites of many species may involve the carbon or amino groups of Gln, Ile, and Arg residues [35]. In the *T. leucocephalus* genome, many PSGs annotated to ion binding have mutations that result in the substitution of Gln, Ile, and Arg residues. For example, the PDLIM3 protein contains a PDZ domain and a LIM domain. PDZ domain proteins have pivotal roles in intestinal anion secretion and salt absorption [36]. In this study, ten positively selected loci in this gene had mutations substituting Gln, Ile, and Arg. Additionally, these mutations can reduce the ion binding affinity of the protein. Some PSGs also had a mutation leading to the insertion of amino acid residues. For example, *SCN8A* is a positively selected locus within *F341I*.

Minerals such as Mg^2+^ and Ca^2+^ are required for a plethora of metabolic processes and signaling pathways. In the genome of *T. leucocephalus*, most genes were expressed in these pathways. FTH1 and FTL, two subunits of the heavy and light ferritin chains, are major iron storage proteins and play key roles in iron metabolism [18]. The gene families encoding these two proteins were expanded in the genome of *T. leucocephalus*, and most copies were expressed in the tissues measured. The blood concentration of Fe^2+^ in *T. leucocephalus* was not significantly different from that in other primates, indicating that this species could effectively process Fe^2+^.

Additionally, *T. leucocephalus* and *T. francoisi* have been reported to sleep in caves on cliff faces which could prevent predation [37]. These caves are typically oxygen-deficient, and the expansion of the *FTH1* and *FTL* gene families could enhance oxygen metabolism. The blood Mg^2+^ concentrations of *T. leucocephalus* were significantly higher than those of the other primates studied. Magnesium is involved in many biochemical and physiological processes, but excessive Mg^2+^ levels could damage health. The *TRPM6* gene is essential for Mg^2+^ uptake in vertebrate cells and modifies Mg^2+^ homeostasis [38]. In the genome of *T. leucocephalus*, four *TRPM6* copies were found and all were expressed. These results indicate that the mineral absorption pathway plays an essential role in adaptation to highly alkaline conditions.

Imbalances of mineral metabolism affect the function of many tissues, such as the nervous system, blood vessels, bone development, and immune function [39–41]. Three nervous system-related terms were enriched in the GO database: “neuronal cell body,” “nervous system development,” and “positive regulation of neuron differentiation” (one gene in TRI and one gene in PGI; all false discovery rate-adjusted *P* < 0.05, Fisher’s exact test; Tables S13, S14, S15). It is reasonable to hypothesize that these genes found in *T. leucocephalus* serve to stabilize the nervous system under high blood concentrations of alkaline minerals. For example, neuronal firing is a fundamental element of cerebral function. The *KCNC2* gene encodes components of voltage-gated potassium channels, which can regulate neuronal firing through the repolarization of action potentials [42]. In *T. leucocephalus*, the expanded orthologous gene families are expressed in many tissues and are enriched in GO database terms related to ion channel binding (Table S14). Thus, our findings suggest that *T. leucocephalus* is adapted to a naturally high mineral ion intake through food and water that is rich in mineral ions [5, 43].

Calcium plays an essential role in the regulation of vital cellular and tissue functions. The concentration of body Ca^2+^ is controlled by Ca^2+^-transporting subsystems (e.g., bones and kidneys) and Ca^2+^-sensing and regulating hormones [44]. High intracellular Ca^2+^ concentrations can also cause toxicity. For example, the transportation of nuclear pore complexes is suppressed at high Ca^2+^ levels [45]. Lui et al. [8] revealed 56 PSGs that are private to *T. leucocephalus* and the four other investigated limestone langur species, and these PSGs are enriched in the Ca^2+^ signaling pathway. Additionally, in vitro investigation indicates that a single point mutation (Lys1905Arg) in the α1c subunit of the L-type voltage-gated calcium channel Ca v 1.2 (CACNA1C) can decrease the inward calcium current into the cells. Many expanded gene families, particular genes, and PSGs that were identified in the *T. leucocephalus* genome (Tables S13–S15) are related to calcium regulation. These had four main functions, namely calcium ion binding, transmembrane transport, voltage-dependent anion channel creation, and cellular response to calcium. The calcium signaling pathway is essential for the regulation of calcium metabolism. In the *T. leucocephalus* genome, four significantly expanded gene families (*VDAC1*, *VDAC2*, *CALM2*, and *SLC25A5*; Fig. 3a and Table S18) and three PSGs (*ADCY1*, *CACNA1G*, and *CCKBR*; false discovery rate-adjusted *P* < 0.05) are related to calcium metabolism. Therefore, the *T. leucocephalus* genome contains adaptations to the high calcium levels found in their karst limestone habitat.

## Conclusion

In this study, we used PacBio sequencing and optimal assembly with Hi-C to obtain a high-quality *T. leucocephalus* genome. Genome-wide transcriptomes of 12 tissues from three *T. leucocephalus* individuals were obtained. This genome provides new phylogenetic and functional insights into primate evolution and helps to elucidate the genetic adaptations of *T. leucocephalus* to highly alkaline environments. The genome annotation and structure prediction revealed that many expanded gene families, particular genes, and PSGs serve to enhance mineral ion binding. Enrichment of the metabolism network revealed that many expanded genes enhance mineral absorption and calcium signaling. This genome enhances our understanding of the unique physiological characteristics of *T. leucocephalus*. Our data provide a resource for analyzing the relationship between the *T. leucocephalus* genome and its habitat and highlight the unique adaptations this species has evolved for persistence in a highly alkaline environment.

## Materials and methods

### De novo library preparation and sequencing

High-molecular-weight genomic DNA was extracted from the peripheral blood of *T. leucocephalus* bred in the Terrestrial Wildlife Rescue and Epidemic Disease Surveillance Center in Guangxi, China, and a *T. francoisi* bred in the Nanning Zoo of Guangxi, China, using a QIAamp DNA Mini Kit and a DNeasy Plant Mini Kit (QIAGEN), following the manufacturer’s instructions. DNA integrity was determined using an Agilent 4200 Bioanalyzer (Agilent Technologies, Palo Alto, CA, USA). The *T. leucocephalus* genome was constructed through single-molecule real-time (SMRT) sequencing using technology developed by PacBio (Pacific BioSciences of California, Inc., Menlo Park, CA, USA) and an Illumina platform (Illumina, San Diego, CA, USA). To do so, 8 μg of genomic DNA was sheared using g-TUBES (Covaris) and concentrated with AMPure PB magnetic beads. SMRTbell libraries were constructed using a PacBio SMRTbell template prep kit 2.1 following the manufacturer’s manual. The constructed libraries were size selected for molecules ~ 20 kb on a BluePippin™ system (Sage Science, Beverly, MA, USA). Primers were annealed, and SMRTbell templates were bound to polymerases using a DNA/polymerase binding kit. Sequencing was performed on a *PacBio RS* II for 10 h. The *T. francoisi* genome was also sequenced using the same methods. Four paired-end and mated-pair sequencing libraries were constructed following Illumina’s protocol.

We combined PacBio sequencing libraries with high-throughput chromosome conformation capture (Hi-C) sequencing libraries for improved scaffold construction. Hi-C library preparation was performed following the standard procedure. In brief, nuclear DNA from the blood was crosslinked in situ, extracted, and digested using a restriction enzyme. The sticky ends of the digested fragments were biotinylated, diluted, and randomly ligated. The biotinylated DNA fragments were enriched and sheared to construct the sequencing library. Sequencing was performed on an Illumina HiSeq platform with paired-end 150-bp reads. Annoroad Gene Technology Co. Ltd. conducted all the Hi-C library preparation. Bowtie 2 [46] and Hi-C-Pro (v2.7.8) [47] were used to align the Hi-C sequencing data to the assembled contigs, and LACHESIS [33] was used to cluster the contigs onto chromosomes (http://shendurelab.github.io/LACHESIS/). A heatmap was constructed for validation.

### Genome assembly and quality assessment

For most genomes, a pure third-generation sequencing assembly strategy (e.g., PacBio) is superior to both second-generation sequencing (i.e., next-generation sequencing) and hybrid assembly that combines second- and third-generation sequencing. Therefore, second-generation sequencing data with 100× coverage were used for survey analysis, and data with 60× coverage were used for genome assembly, corrected by second-generation sequencing data. *k*-mer analysis was performed using Jellyfish (v2.2.0) [48] to predict the size of the *T. leucocephalus* genome. The three main steps were carried out to obtain high-quality subreads (corrected using the parameters corOutCoverage = 80 and core concurrency = 20). First, all-vs-all overlaps were constructed using a *k*-mer histogram following the indexed store of input subreads. The best overlaps were utilized for correction and corrected subread generation. Second, the Falcon (https://github.com/cschin/Falcon) and SMARTdenovo software [49] were used to assemble a more continuous draft genome. The use of the SMARTdenovo program involved five steps. The Wtpre program was used to prepare the corrected subreads (using the parameter -J 5000). Then, homopolymer-compressed *k*-mer seeding was employed to identify overlapping long reads using the wtzmo program (parameters -k 16, -z 10, -Z 16, -U -1, -m 0.1, and -A 1000). Long reads were clipped via the wtclp program (parameters -d 3, -k 300, -m 0.1, and -FT) to maximize the legal overlap results, and wtlay was employed for layout construction based on the best overlap graph result (parameters -w 500, -s 100, -m 0.1, -r 0.85, and -c 1). Next, wtcns was used to obtain high-quality consensus sequences as the final draft genome. Raw subreads were mapped back to the consensus sequences using blast (SmartLink 5.0) to improve the accuracy of consensus sequences. Arrow (SmartLink 5.0) was used to polish consensus sequences with default parameters. Pilon (v1.22) [13] was utilized to correct the polished contigs with default parameters and to improve the local base accuracy of the contigs assembled through Illumina reads. Three approaches were employed to evaluate the quality of the genome following assembly. First, blood RNA-Seq was assembled to format a high-quality cDNA/EST using Trinity. The EST was mapped onto the assembled genome to assess the integrity of the gene coding region. Second, BUSCO (v3.0) [50] was applied as an evolutionary measure of genome completeness using mammalia_odb9 (4104 genes) as the query. Lastly, Illumina DNA-Seq was mapped onto the genome to calculate the mapping ratio of the reads and the degree and depth of genome coverage.

SOAPDenovo2 (v2.04) [51] was employed to construct the contigs and scaffolds of the *T. francoisi* genome (with the parameter –K 51). In brief, contigs were first de novo assembled with two short insert library reads. Then, all of the reads were aligned to construct scaffolds with the following two mate-paired reads. The assembled genome was subjected to gap closing using GapCloser (v1.12; https://sourceforge.net/projects/soapdenovo2/files/GapCloser) with default parameters. The 350-bp insert library reads were used to detect the genome mapping rate and coverage. BUSCO was also used to evaluate the *T. francoisi* genome.

### Repeat annotation

Repetitive sequences and transposable elements (TEs) in the genome were identified using a combination of de novo and homology-based approaches. RepeatMasker (open-4.0.6) and RepeatProteinMask (v.4.0.6) [15] were used to identify and classify different repeats with TEs and short tandem repeats by aligning *T. leucocephalus* genome sequences against the Repbase database (RepBase23.12) [52] with default parameters. TRF (v4.0.6) [53] was used (with the parameters Match = 2, Mismatch = 7, Delta = 7, PM = 80, PI = 10, Minscore = 50, MaxPerid = 500, -d, -h) to identify tandem repeats.

### Genome annotation

Protein-coding regions were identified through a combination of homology, de novo, and transcriptome-based prediction methods. Homologous protein sequences from several species, including *H. sapiens*, *M. mulatta*, *R. bieti*, and *R. roxellana*, were downloaded from NCBI. These protein sequences were mapped against those of *T. leucocephalus* using tblastn (v2.2.28+). Blast hits were linked to candidate gene location using solar (v0.1.20; parameters -a prot2genome2 and –z). GeneWise (v2.2.0) [54] was employed to extract candidate gene loci, including 1000 bp of upstream and downstream flanking DNA, and to define intron–exon boundaries. Genes with a length > 150 bp or with the correct structure (premature stop codon or frameshifts) were selected for further analysis. De novo transcriptome assembly was performed using Trinity. The assembled transcripts were passed to PASA to exploit the spliced alignments of the expressed transcript sequences and model the gene structures automatically. Ab initio predictions were made using AUGUSTUS (v3.3) [54] and GeneMark-ES (v4.32) [55] with default parameters. The final weighted consensus gene structure was constructed using EVidenceModeler (v1.1.1) [56] with default parameters.

After the genes were modeled, functional annotation was performed by subjecting protein sequences to a BLASTP search against several databases, including Nt, Pfam, Swiss-Prot, KEGG, and GO, with an *E* value of 1E−5. Protein domains were annotated by mapping to the Pfam database using HMMER (v3.1b1) [57]. A Trinotate pipeline was utilized to identify the pathways and GO terms associated with genes.

### Noncoding RNA annotation

Noncoding RNA (ncRNA) is a class of RNAs that are not translated into proteins. We identified four types of ncRNA: miRNAs, tRNAs, rRNAs, and snRNAs. tRNA genes were identified by applying tRNAscan-SE (v1.3.1) [58] with default parameters. rRNA fragments were predicted by aligning human rRNA sequences to *T. leucocephalus* sequences using BLASTN with a parameter of *E* value <1E−5. miRNA and snRNA genes were searched with BLAST against the Rfam (v13.0) database using INFERNAL (v1.0) with Rfam’s family-specific “gathering” cut-off [59].

### Genetic family and phylogenetic analysis

Sequences from 14 other mammals, namely *H. sapiens*, *C. atys*, *C. sabaeus*, *C. angolensis*, *M. fascicularis*, *M. mulatta*, *M. nemestrina*, *N. leucogenys*, *P. paniscus*, *P. troglodytes*, *P. anubis*, *P. tephrosceles*, *R. bieti*, and *R. roxellana*, were downloaded from NCBI. These sequences, along with *T. leucocephalus* and *T. francoisi* sequences, were utilized to reconstruct a phylogenetic tree using a maximum likelihood algorithm. All-vs-all BLAST was performed for all protein sequences with the following thresholds: *E* value <10E−10 and identity > 30%. Gene families were adopted by applying the hcluster_sq software of TreeFam (http://www.treefam.org). Orthologous protein sequences were aligned using MUSCLE (v3.6) [60]. The conserved regions were used to construct a maximum likelihood tree in PhyML (v3.0) [61].

### Divergence time estimation

Divergence times were estimated based on a set of fourfold degenerate sites from amino acids that are conserved across all mammals using the coding sequences of 5345 single-copy orthologous genes. The MCMCTREE (v4.5) model in PAML [62] was used to estimate divergence times based on phylogenetic relationships, calibrated with the divergence time between *H. sapiens* and *P. troglodytes* (6.23–7.07 Mya) [63], selected from www.timetree.org. MCMCTREE was parameterized to sample 10,000 times, with a sampling frequency of 5000 after a burn-in of 5,000,000 iterations. Default settings were used for the other parameters.

### Segmental duplication

Segmental duplications (SDs) are duplicated blocks of genomic DNA that typically range in size from 1 to 200 kb. Genomic regions with alignment lengths exceeding 1 kb and identities from 90 to 98% were identified as SDs based on self-genome alignments in LASTZ (v1.02.00; https://github.com/lastz/lastz). Repeat-masked genome sequences were subjected to self-vs-self alignment using LASTZ (v1.04). Segmental duplication sequences were identified with nonrepeat alignment lengths > 500 bp and identities > 85%. The repeat-masked regions of genome sequences were reintroduced for optimal global alignment to more accurately refine the identity and define the boundaries of the segment.

### Analysis of gene family expansion and contraction

The expansion and contraction of gene families were determined by comparing cluster sizes between the genomes of the ancestor and those of *T. leucocephalus* and 13 other mammals using the CAFE [63] program (http://sourceforge.net/projects/cafehahnlab/). In CAFE, a random birth and death model was used to check for changes in each gene family in accordance with each lineage in the tree. A probabilistic graphical model was introduced to calculate the probability of transitions in the gene family size from parent to child nodes in a tree. The possible *P* value in each lineage was calculated in accordance with conditional likelihoods. A *P* value of 0.05 was used to identify significantly expanded gene families.

### Positive selection analysis

Orthologous genes among *T. leucocephalus* and 14 other mammal species, namely *H. sapiens*, *C. atys*, *C. sabaeus*, *C. angolensis*, *M. fascicularis*, *M. mulatta*, *M. nemestrina*, *N. leucogenys*, *P. paniscus*, *P. troglodytes*, *P. anubis*, *P. tephrosceles*, *R. bieti*, and *R. roxellana*, were identified using an all-vs-all BLAST search. A total of 5345 orthologous family sequences were aligned using PRANK [64] with default parameters. Branch-site likelihood ratio tests were used to detect positive selection based on branch-site models in the PAML software. *P* values were computed via the *χ*^2^ statistics. Significant PSGs with *P* < 0.05 were annotated using various databases, such as Nt, Nr, KEGG, Swiss-Prot, and GO.

### Transcriptome sequencing and analysis

Six tissues were isolated from the lungs, livers, testes, muscles, kidneys, and hearts of two white-headed male langurs that died from traffic injury in the Guangxi Chongzuo White-Headed Langur National Nature Reserve in Guangxi province, China. Peripheral blood was obtained from a white-headed langur male bred in the Terrestrial Wildlife Rescue and Epidemic Disease Surveillance Center of Guangxi. Total RNA was extracted using the TRIzol method. Libraries were constructed using a standard Illumina mRNA-Seq Prep Kit and sequenced on an HiSeq X Ten PE150 platform. Clean transcriptome reads were mapped to the *T. leucocephalus* genome using HISAT2 [65]. The Htseq-count [66] was used to generate a count of the reads mapped to each gene for calculation at the gene level. Count results were used to further generate the number of fragments per kilobase per million mapped fragments (FPKM) [67] via custom scripts.

### Structural variant identification

Because the closest relationship was found between *T. leucocephalus* and *T. francois*, we constructed a chromosome genome for *T. francois* using RaGOO (v1.1, https://github.com/malonge/RaGOO) with default parameters. This method could also detect structural variants (SVs) between these two species. We used the same method to identify SVs between *M. mulatta*, *T. leucocephalus*, and *T. francoisi* (three pairwise comparisons)*.*

### Metagenomic scanning of 21 fecal samples of wild *T. leucocephalus*

#### Sample collection

Fresh fecal samples were collected from 20 *T. leucocephalus* individuals at the Guangxi Chongzuo White-Headed Langur Nature Reserve and one captive *T. leucocephalus* at the Terrestrial Wildlife Rescue and Epidemic Disease Surveillance Center of Guangxi. Immediately after the wild *T. leucocephalus* defecated, the feces were collected and placed on dry ice at − 70 °C, shipped to the laboratory, and stored at − 80 °C until DNA extraction.

#### DNA extraction

Total DNA was extracted from fecal samples using a PowerSoil DNA Isolation Kit (MoBio, Solana Beach, CA, USA) following the manufacturer’s instructions. Agarose gel electrophoresis was conducted to assess the quality of DNA. OD was 1.8–2.0, and DNA concentrations above 1 μg were used to construct the library.

#### DNA library construction

DNA (1 μg per sample) was used for library construction. Sequencing libraries were generated using the NEBNext® Ultra™ DNA Library Prep Kit for Illumina (New England BioLabs, Inc., USA) following the manufacturer’s instructions [68], and index codes were added to uniquely identify each sample. The DNA was then fragmented to a size of 350 bp through sonication, and fragments were end-polished, A-tailed, and ligated with a full-length adaptor for Illumina sequencing and further PCR amplification. PCR products were purified (AMPure XP), library size distributions were analyzed using an Agilent 2100 bioanalyzer, and libraries were quantified through real-time PCR.

#### Sequencing

Index-coded samples were clustered on a cBot Cluster Generation System following the manufacturer’s instructions. After a cluster was made, the prepared libraries were sequenced on an Illumina HiSeq platform and paired-end reads were generated [69].

#### De novo assembly and data analysis

Raw reads that met certain conditions were trimmed to obtain high-quality clean reads and thereby improve the reliability of data processing. SoapAligner (v2.21; identity ≥90%, -l 30, -v 7, -M 4,-m 200, -x 400) [69] was used to remove the *T. leucocephalus* genome. The clean data were then subjected to de novo assembly using SOAPdenovo (V2.21) [51]. For each sample, *k*-mer = 55 was selected for the assembly (with the parameters -d 1, -M 3, -R, -u, and -F), and scaffolds were obtained. The scaffolds were interrupted from the N joint to obtain scaftigs. Then, SoapAligner was used to BLAST the clean data to the scaftigs of each sample and acquire paired-end reads. Paired-end reads were placed together, and those with *k*-mer = 55 were selected for compounded assembly. The assemblies were interrupted from the N joint and the scaftig that did not contain N was obtained to compound the assembly results. Scaftigs shorter than 500 bp were not included in the subsequent statistical analysis and gene prediction.

#### Gene prediction

MetaGeneMark (version 2.10) [70] was used to predict ORFs and filter information with lengths < 100 bp. Redundancies were eliminated using the Cluster Database at High Identity with Tolerance [71] program to obtain the initial nonredundant gene catalog (with the parameters -c 0.95, -G 0, -aS 0.9, -g 1, and -d 0). SoapAligner was used to align clean data (with parameters -m 200, -x 400, and identity ≥95%). Genes with several reads ≤2 were filtered to obtain a gene catalog for subsequent analysis. Unigenes were aligned against the database, including bacteria, fungi, archaea, and viruses, which were extracted from the NCBI-nr database using DIAMOND. The MEGAN (v4, Tübingen, Baden-Württemberg, Germany) [72] software was utilized to obtain the abundance information and gene count table of each sample at each classification level.

#### Gene function prediction

BLASTp was used to search the protein sequences of the predicted genes in the eggNOG and KEGG databases with *E* value <1E−5. Unigene sequences were searched in the Antibiotic Resistance Genes Database (ARGD) [39] with *E* value ≤1E−5 to identify antibiotic resistance genes (ARGs), antibiotic resistance type, and the antibiotics that the ARGs could tolerate.

## Supplementary Information

**Additional file 1:** **Figure S1.** The scaffold sequence and expression of PCDHA6 and PCDHGA9 families. A: The scaffold of PCDHA6 genes in the T. leucocephalus genome. B: The expression of all the PCDHA6 families. C: The scaffold of PCDHGA9 genes in the T. leucocephalus genome. D: The expression of all the PCDHGA9 families.

**Additional file 2:** **Figure S2.** The positively selected locus in site 841 of SCN8A. A: The amino sequence around the mutation F841I with the red marker. B. The residue information about SCN8A is predicted by the expasy database (https://swissmodel.expasy.org/). C. The 3D structure of SCN8A is predicted by the expasy database (https://swissmodel.expasy.org/). The arrow indicates the mutation F841I.

**Additional file 3:** **Figure S3.** The evolution and expression of FTH1 families. A: The evolution of FTH1 families for 15 primates. B: The expression of FTH1 families about 15 primates.

**Additional file 4:** **Figure S4.** Four mineral ion concentration in the blood between Limestone langurs and other primates. A: The blood Mg2+ concentrations for five primate groups. B: The blood potassium concentrations for six primate groups. C: The blood sodium concentrations for six primate groups. D: The blood Ca2+ concentrations for six primate groups.

**Additional file 5:** **Figure S5.** the characters of expanded VDACs families in the T. leucocephalus genome. A: The expression of VDAC1/2 families. B: the evolution of VDAC1 families. C: the evolution of VDAC2 families.

**Additional file 6:** **Table S1.** Illumina libraries used in the de novo sequencing of the T. leucocephalus genome.

**Additional file 7:** **Table S2.** Survey statistics for the T. leucocephalus genome.

**Additional file 8:** **Table S3.** T. leucocephalus genome assembly statistics.

**Additional file 9:** **Table S4.** Statistics for Illumina reads mapped to the T. leucocephalus genome.

**Additional file 10:** **Table S5.** Statistics for EST mapped to the T. leucocephalus genome.

**Additional file 11:** **Table S6.** Hi-C statistics for the chromosome genome assembly.

**Additional file 12:** **Table S7.** Summary of the functional gene annotation of T. leucocephalus.

**Additional file 13:** **Table S8.** Summary of non-coding RNAs for T. leucocephalus.

**Additional file 14:** **Table S9.** Summary of TE for T. leucocephalus.

**Additional file 15:** **Table S10.** The detailed information (version, website links, etc.) of genome assemblies of 14 other primates used in this study.

**Additional file 16:** **Table S11.** Summary of the repeat TE classes for T. leucocephalus.

**Additional file 17:** **Table S12.** Top 10 enrichment pathways of the special gene families of T. leucocephalus.

**Additional file 18:** **Table S13.** The annotation of special genes in the genome of T. leucocephalus.

**Additional file 19 :** **Table S14.** The annotation of expanded gene families in the genome of T. leucocephalus.

**Additional file 20:** **Table S15.** The annotation of contracted gene families in the genome of T. leucocephalus.

**Additional file 21:** **Table S16.** Annotation analysis of positive selected genes related to alkaline ion metabolism of T. leucocephalus.

**Additional file 22:** **Table S17.** The expression of all genes in mineral absorption pathways from seven tissues of T. leucocephalus.

**Additional file 23:** **Table S18.** Annotation analysis of positive selected genes related to alkaline ion metabolism of T. leucocephalus.

## Data Availability

Raw sequence reads of the *T. francoisi* genome have been deposited in the Genome Sequence Archive (Genomics, Proteomics & Bioinformatics 2017) at the Beijing Institute of Genomics Data Center (Nucleic Acids Res 2019), Chinese Academy of Sciences, under accession number CRA002266(publicly accessible at https://bigd.big.ac.cn/gsa).
