# Supplementary figures and images for: The reference genome and transcriptome of the limestone langur, Trachypithecus leucocephalus, reveal expansion of genes related to alkali tolerance
Source: BMC Biol. 2021 Apr 8;19:67. doi: 10.1186/s12915-021-00998-2 (PMC8034193; doi:10.1186/s12915-021-00998-2)

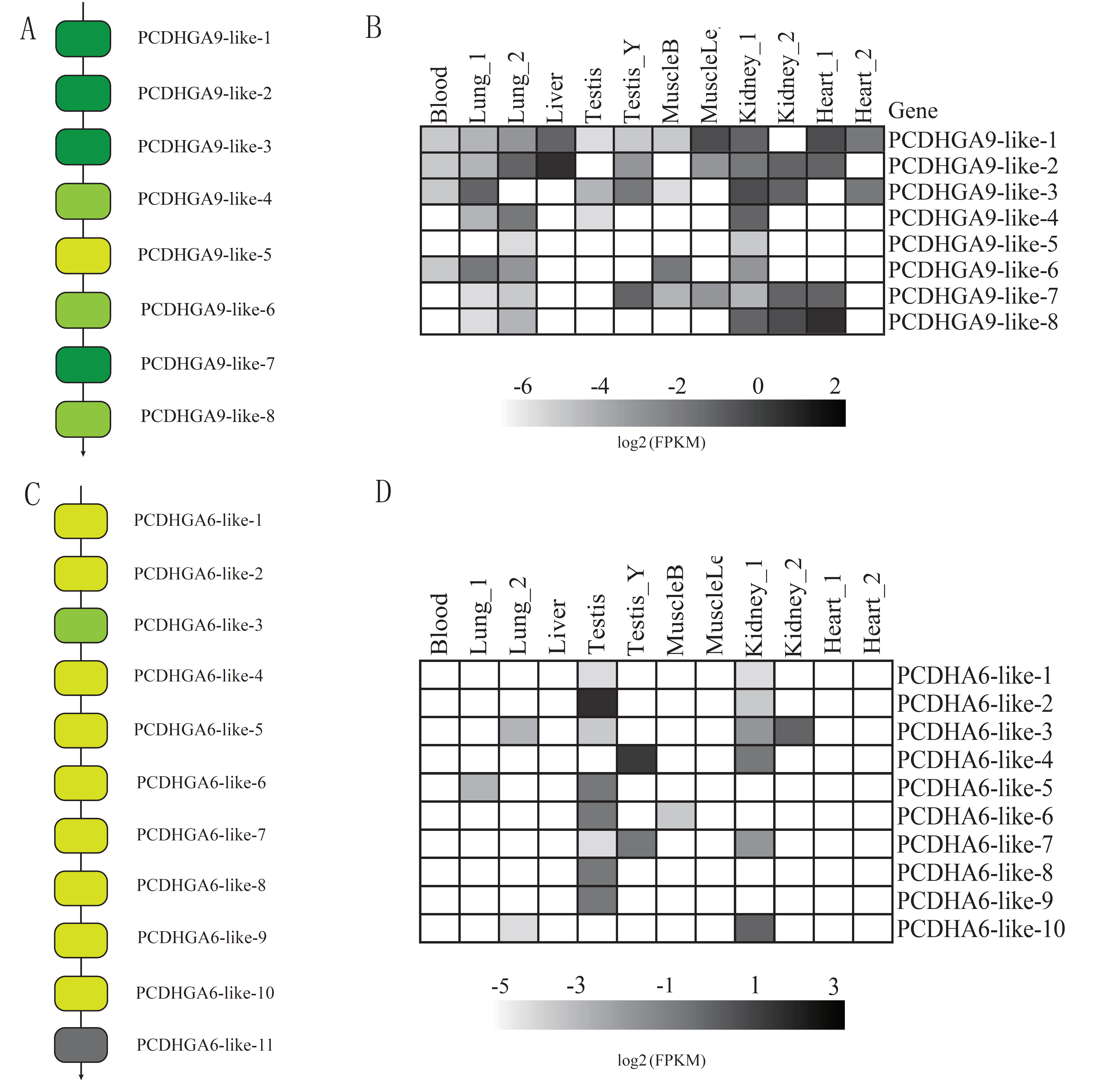

Supplement: Supplementary file 1 — Additional file 1: Figure S1. The scaffold sequence and expression of PCDHA6 and PCDHGA9 families. A: The scaffold of PCDHA6 genes in the T. leucocephalus genome. B: The expression of all the PCDHA6 families. C: The scaffold of PCDHGA9 genes in the T. leucocephalus genome. D: The expression of all the PCDHGA9 families. [file 12915_2021_998_MOESM1_ESM.jpg]

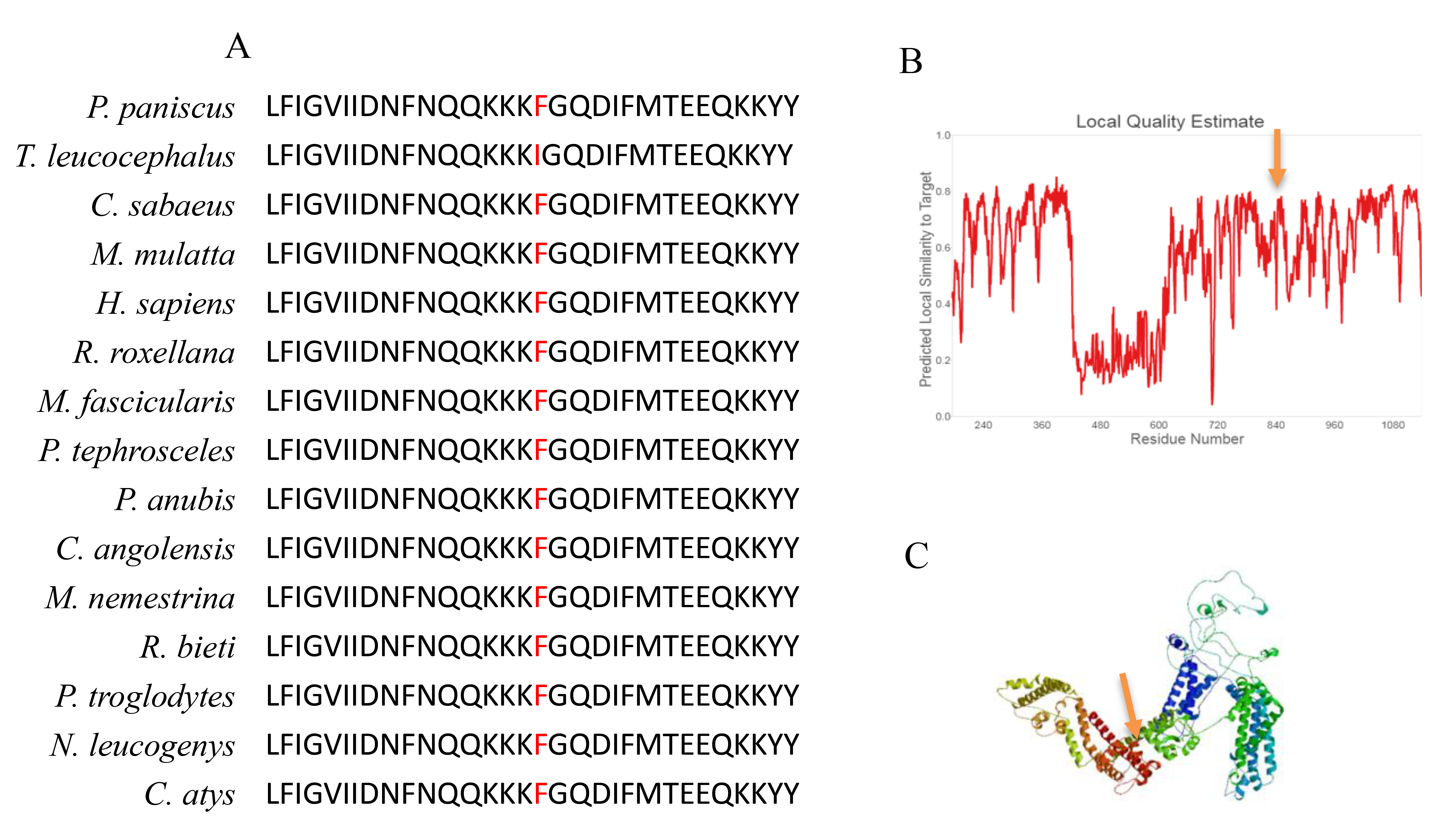

Supplement: Supplementary file 2 — Additional file 2: Figure S2. The positively selected locus in site 841 of SCN8A. A: The amino sequence around the mutation F841I with the red marker. B. The residue information about SCN8A is predicted by the expasy database (https://swissmodel.expasy.org/). C. The 3D structure of SCN8A is predicted by the expasy database (https://swissmodel.expasy.org/). The arrow indicates the mutation F841I. [file 12915_2021_998_MOESM2_ESM.jpg]

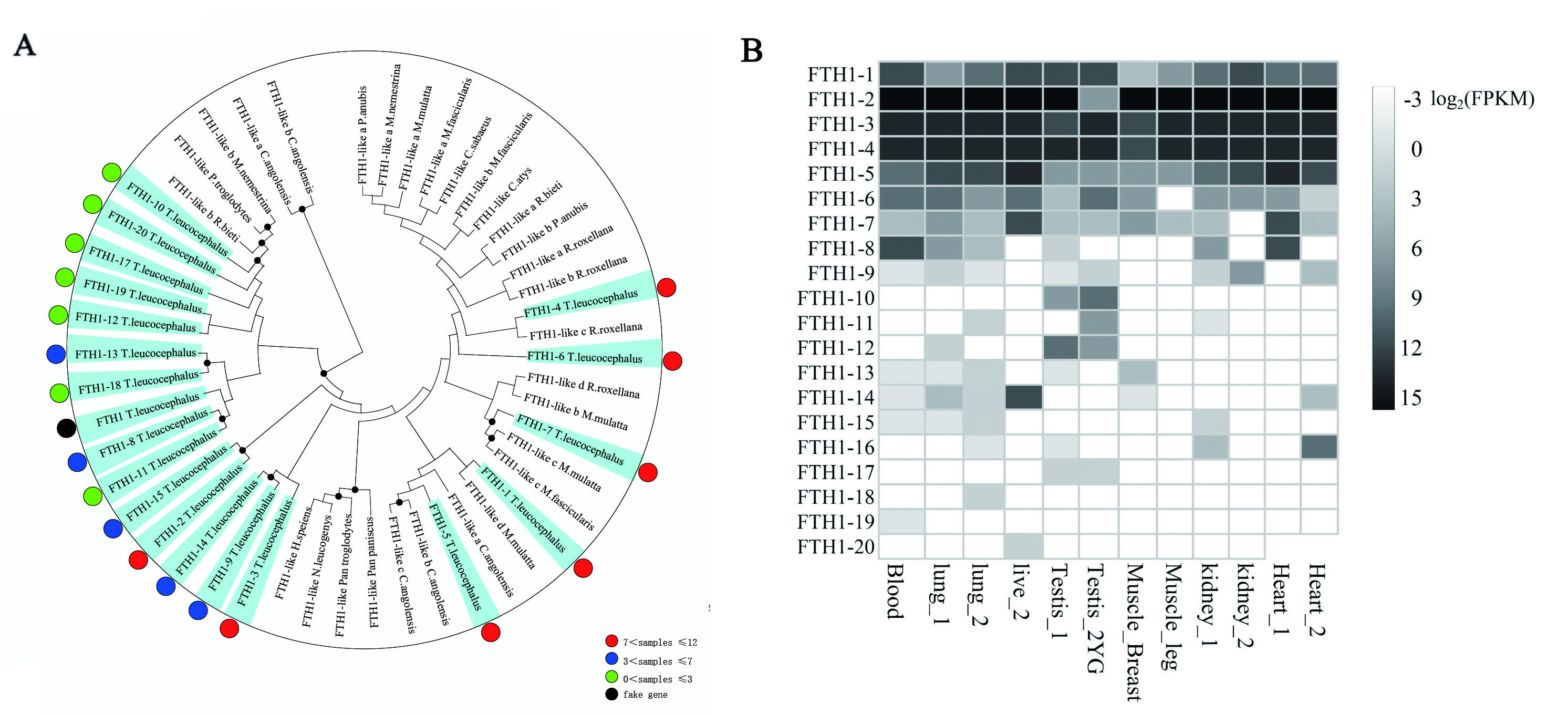

Supplement: Supplementary file 3 — Additional file 3: Figure S3. The evolution and expression of FTH1 families. A: The evolution of FTH1 families for 15 primates. B: The expression of FTH1 families about 15 primates. [file 12915_2021_998_MOESM3_ESM.jpg]

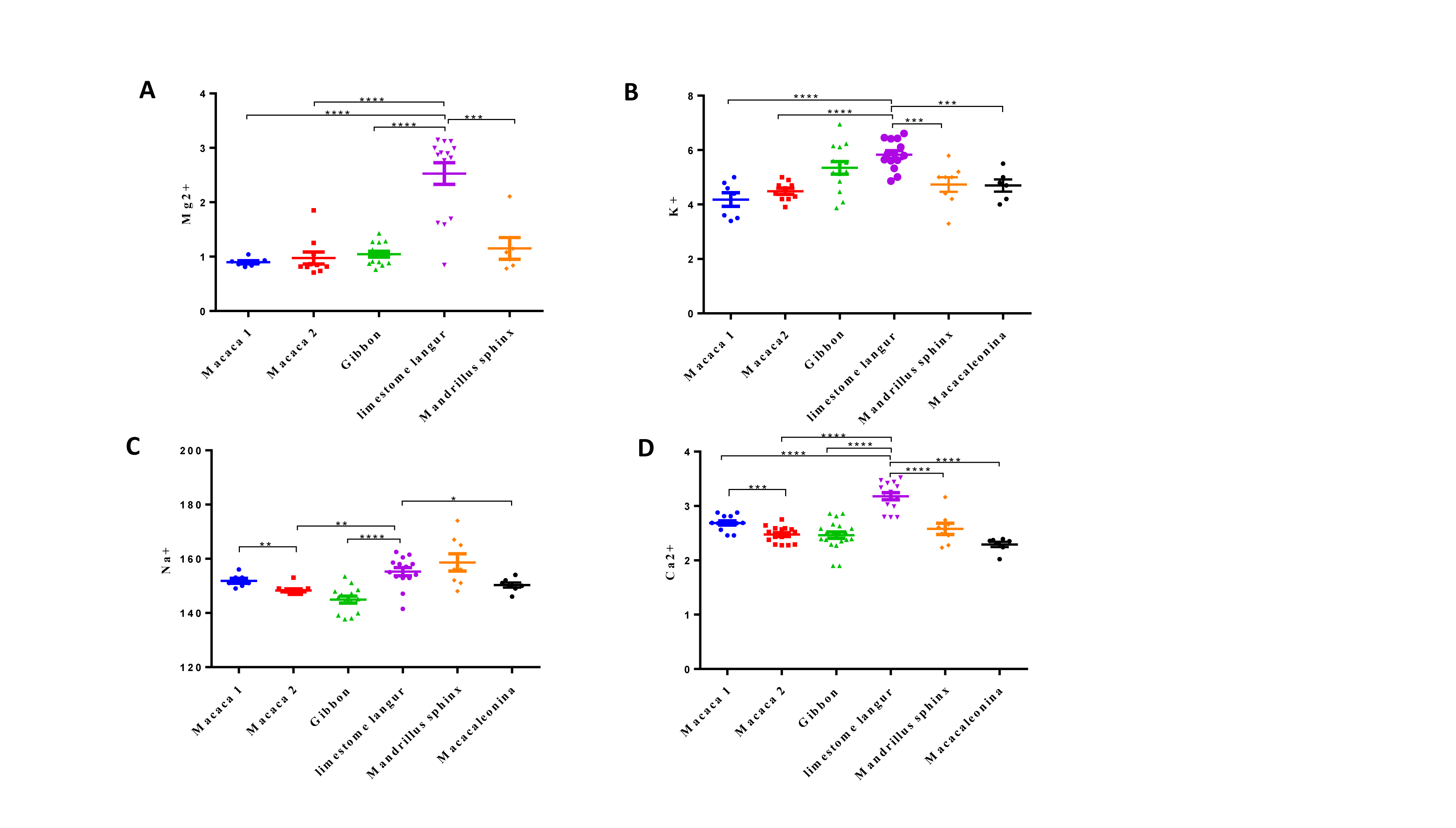

Supplement: Supplementary file 4 — Additional file 4: Figure S4. Four mineral ion concentration in the blood between Limestone langurs and other primates. A: The blood Mg2+ concentrations for five primate groups. B: The blood potassium concentrations for six primate groups. C: The blood sodium concentrations for six primate groups. D: The blood Ca2+ concentrations for six primate groups. [file 12915_2021_998_MOESM4_ESM.jpg]

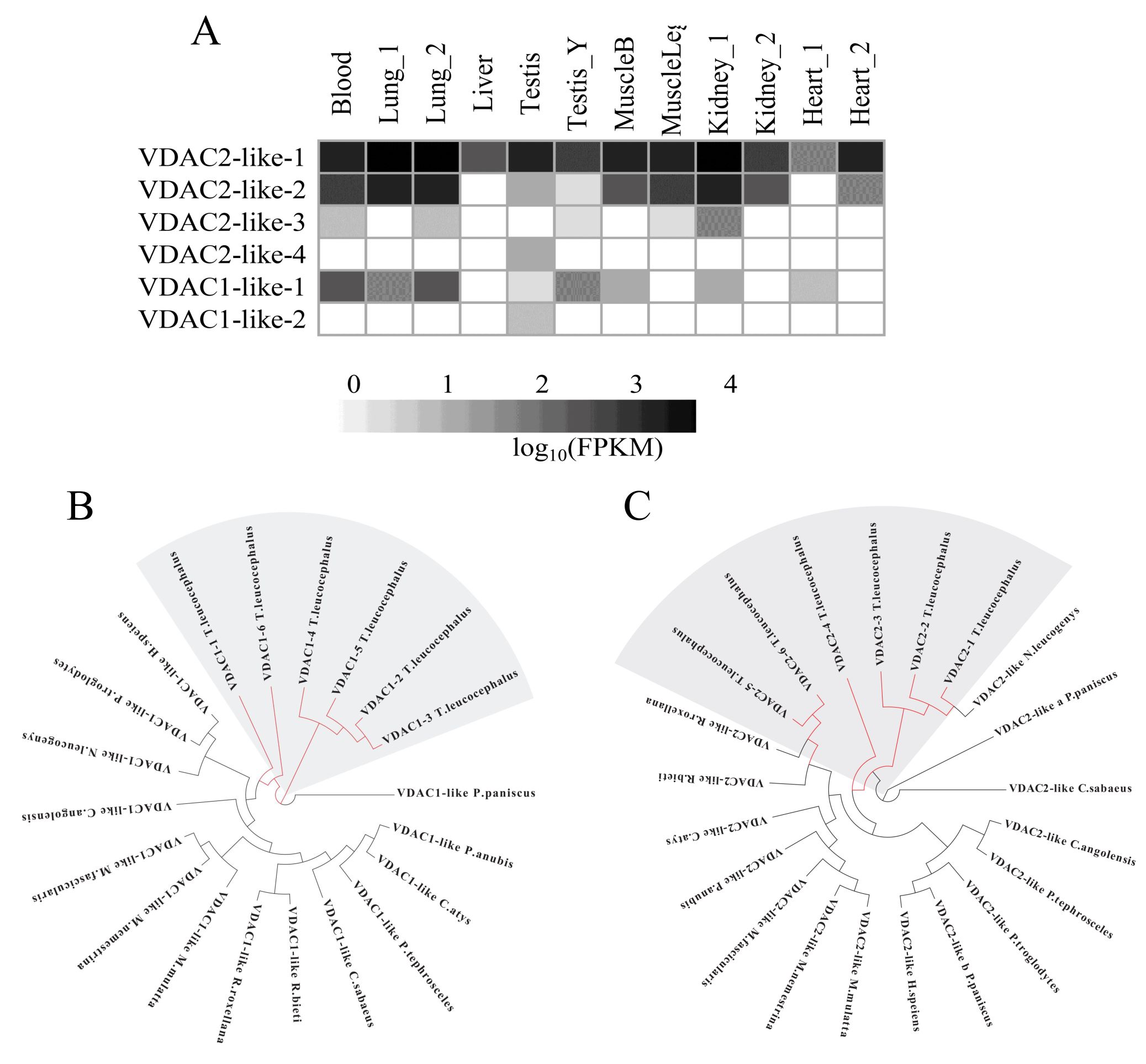

Supplement: Supplementary file 5 — Additional file 5: Figure S5. the characters of expanded VDACs families in the T. leucocephalus genome. A: The expression of VDAC1/2 families. B: the evolution of VDAC1 families. C: the evolution of VDAC2 families. [file 12915_2021_998_MOESM5_ESM.jpg]
