# Supplementary material for: The reference genome and transcriptome of the limestone langur, Trachypithecus leucocephalus, reveal expansion of genes related to alkali tolerance
Source: BMC Biol. 2021 Apr 8;19:67. doi: 10.1186/s12915-021-00998-2 (PMC8034193; doi:10.1186/s12915-021-00998-2)
Supplement: Supplementary file 6 — Additional file 6: Table S1. Illumina libraries used in the de novo sequencing of the T. leucocephalus genome. [file 12915_2021_998_MOESM6_ESM.docx]

| **Additional file 6: Table S1: Illumina libraries used in the de novo sequencing of the T. leucocephalus genome.** | | | | | | |
| --- | --- | --- | --- | --- | --- | --- |
| Species name | Insert size (bp) | Read length | Raw reads | | Clean reads^a^ | |
|  |  |  | Total data (Gb) | Physical coverage (X)^b^ | Total data (Gb) | Physical coverage (X)^b^ |
| *T. leucocephalus* | 500 | 150 | 197 | 69.1 | 170 | 59.6 |
|  |  |  |  |  |  |  |
| a Qualified reads were collected by filtering low-quality reads (the rate of quality of base < 19 is more than 50%), single read, base-calling duplicates, and adapter bases with more than 5 bp and more than 5% N contamination from the raw data. | | | | | | |
| b Coverage was calculated under assumed genome sizes of 2.85 Gb for T.leucocephalus and 2.94 Gb for T.francoisi. Physical coverage refers to the total length of clean reads relative to genome size. | | | | | | |
