# Supplementary material for: The reference genome and transcriptome of the limestone langur, Trachypithecus leucocephalus, reveal expansion of genes related to alkali tolerance
Source: BMC Biol. 2021 Apr 8;19:67. doi: 10.1186/s12915-021-00998-2 (PMC8034193; doi:10.1186/s12915-021-00998-2)
Supplement: Supplementary file 7 — Additional file 7: Table S2. Survey statistics for the T. leucocephalus genome. [file 12915_2021_998_MOESM7_ESM.docx]

**Additional file 7: Table S2: Survey statistics for the T. leucocephalus genome.**

| Sample | K-mer | K-mer number | K-mer depth | Genome size (Mbp) | Revised genome size (Mbp) | Heterozygous ratio (%) | Repeat (%) |
| --- | --- | --- | --- | --- | --- | --- | --- |
| *T.leucocephalus* | 21 | 134,533,905,984 | 48 | 2,802.79 | 2,660.36 | 0.35 | 22.27 |
