# Supplementary material for: The reference genome and transcriptome of the limestone langur, Trachypithecus leucocephalus, reveal expansion of genes related to alkali tolerance
Source: BMC Biol. 2021 Apr 8;19:67. doi: 10.1186/s12915-021-00998-2 (PMC8034193; doi:10.1186/s12915-021-00998-2)
Supplement: Supplementary file 8 — Additional file 8: Table S3. T. leucocephalus genome assembly statistics. [file 12915_2021_998_MOESM8_ESM.docx]

| **Additional file 8: Table S3: T. leucocephalus genome assembly statistics.** | | | | |  |
| --- | --- | --- | --- | --- | --- |
| Stat Type | SMARTDenovo^a^ | | Falcon^b^ | |  |
|  | Contig Length (bp) | Contig Number | Contig Length (bp) | Contig Number |  |
| Total | 2,848,895,678 | 2,612 | 2,811,433,620 | 11,133 |  |
| Max_length | 40,345,259 | - | 4,130,964 | - |  |
| Number>=2kb | - | 2,612 | - | 11,133 |  |
| N50 | 5,646,921 | 150 | 711,652 | 1,122 |  |
| N60 | 4,285,947 | 208 | 529,900 | 1,584 |  |
| N70 | 3,025,841 | 286 | 388,557 | 2,207 |  |
| N80 | 1,922,114 | 405 | 257,564 | 3,088 |  |
| N90 | 742,972 | 635 | 120,806 | 4,639 |  |
|  |  |  |  |  |  |
| a SMARTDenovo was used in the assembly by using the corrected reads from Canu. | | | | |  |
| b Falcon used all raw subreads. The corrected step was performed by self. | | | | |  |
