# Supplementary material for: The reference genome and transcriptome of the limestone langur, Trachypithecus leucocephalus, reveal expansion of genes related to alkali tolerance
Source: BMC Biol. 2021 Apr 8;19:67. doi: 10.1186/s12915-021-00998-2 (PMC8034193; doi:10.1186/s12915-021-00998-2)
Supplement: Supplementary file 9 — Additional file 9: Table S4. Statistics for Illumina reads mapped to the T. leucocephalus genome. [file 12915_2021_998_MOESM9_ESM.docx]

| **Additional file 9: Table S4: Statistics for Illumina reads mapped to the T. leucocephalus genome.** | | | | |
| --- | --- | --- | --- | --- |
| Sample | T. leucocephalus |  |  |  |
| Clean reads | 602,120,674 |  |  |  |
| Clean bases | 90,318,101,100 |  |  |  |
| Mapped bases | 90,085,727,100 |  |  |  |
| Map rate (%) | 99.74 |  |  |  |
| Mean Depth | 31.32 |  |  |  |
| Coverage Rate (%) | 99.65 |  |  |  |
| Coverage at least 4X (%) | 99.33 |  |  |  |
| Coverage at least 10X (%) | 97.97 |  |  |  |
| Coverage at least 20X (%) | 86.59 |  |  |  |
