# Supplementary material for: The reference genome and transcriptome of the limestone langur, Trachypithecus leucocephalus, reveal expansion of genes related to alkali tolerance
Source: BMC Biol. 2021 Apr 8;19:67. doi: 10.1186/s12915-021-00998-2 (PMC8034193; doi:10.1186/s12915-021-00998-2)
Supplement: Supplementary file 10 — Additional file 10: Table S5. Statistics for EST mapped to the T. leucocephalus genome. [file 12915_2021_998_MOESM10_ESM.docx]

| **Additional file 10: Table S5: Statistics for EST mapped to the T. leucocephalus genome.** | | | | | | | |  |  |
| --- | --- | --- | --- | --- | --- | --- | --- | --- | --- |
| Species | Dataset | Number | Total length (bp) | Bases covered by assembly(%) | Sequences covered by assembly (%) | >90% sequence in one scaffold | | with >50% sequence in ont scaffold | |
|  |  |  |  |  |  | Number | Percentage (%) | Number | Percentate (%) |
| *T.leucocephalus* | >0bp | 114,177 | 155,803,225 | 98.77 | 99.66 | 107,591 | 94.23 | 113,548 | 99.45 |
|  | >200bp | 114,177 | 155,803,225 | 98.77 | 99.66 | 107,591 | 94.23 | 113,548 | 99.45 |
|  | >500bp | 65,999 | 140,979,629 | 98.82 | 99.83 | 62,670 | 94.96 | 65,736 | 99.60 |
|  | >1000bp | 45,727 | 126,611,962 | 98.85 | 99.90 | 43,750 | 95.68 | 45,581 | 99.68 |
|  | >2000bp | 26,335 | 98,404,396 | 98.81 | 99.91 | 25,218 | 95.76 | 26,251 | 99.68 |
|  | >5000bp | 4,542 | 30,822,179 | 98.34 | 99.76 | 4,280 | 94.23 | 4,513 | 99.36 |
| *T.francoisi* | >0bp | 114,177 | 155,803,225 | 97.71 | 99.72 | 99,389 | 87.05 | 112,510 | 98.54 |
|  | >200bp | 114,177 | 155,803,225 | 97.71 | 99.72 | 99,389 | 87.05 | 112,510 | 98.54 |
|  | >500bp | 65,999 | 140,979,629 | 97.71 | 99.90 | 56,040 | 84.91 | 64,877 | 98.30 |
|  | >1000bp | 45,727 | 126,611,962 | 97.72 | 99.96 | 38,402 | 83.98 | 44,891 | 98.17 |
|  | >2000bp | 26,335 | 98,404,396 | 97.68 | 99.97 | 21,689 | 82.36 | 25,827 | 98.07 |
|  | >5000bp | 4,542 | 30,822,179 | 97.22 | 99.85 | 3,677 | 80.96 | 4,434 | 97.62 |
