# Supplementary material for: The reference genome and transcriptome of the limestone langur, Trachypithecus leucocephalus, reveal expansion of genes related to alkali tolerance
Source: BMC Biol. 2021 Apr 8;19:67. doi: 10.1186/s12915-021-00998-2 (PMC8034193; doi:10.1186/s12915-021-00998-2)
Supplement: Supplementary file 11 — Additional file 11: Table S6. Hi-C statistics for the chromosome genome assembly. [file 12915_2021_998_MOESM11_ESM.docx]

| **Additional file 11: Table S6: Hi-C statistics for the chromosome genome assembly.** | |
| --- | --- |
| Class | Stat |
| Clean Paired-end Reads | 1,639,349,567 |
| Unmapped Paired-end Reads | 15,695,317 |
| Unmapped Paired-end Reads Rate (%) | 0.96 |
| Paired-end Reads with Singleton | 133,092,789 |
| Paired-end Reads with Singleton Rate (%) | 8.12 |
| Multi Mapped Paired-end Reads | 239,866,406 |
| Multi Mapped Ratio (%) | 14.63 |
| Unique Mapped Paired-end Reads | 1,250,695,055 |
| Unique Mapped Ratio (%) | 76.29 |
| Unique Mapped Paired-end Reads | 1,250,695,055 |
| Dangling End Paired-end Reads | 145,410,170 |
| Dangling End Rate (%) | 11.63 |
| Self Circle Paired-end Reads | 7,460,103 |
| Self Circle Rate (%) | 0.6 |
| Dumped Paired-end Reads | 3,505,954 |
| Dumped Rate (%) | 0.28 |
| Interaction Paired-end Reads | 1,094,318,828 |
| Interaction Rate (%) | 87.5 |
| Valid Paired-end Reads | 1,092,868,162 |
| Valid Rate (%) | 87.38 |
| Number of Sequence in Draft Genome | 2721 |
| Length of Sequence in Draft Genome(bp) | 2,842,413,467 |
| Number of Sequence in Clustering | 1171 |
| Rate of Number in Clustering(%) | 43.04 |
| Length of Sequence in Clustering(bp) | 2,643,208,319 |
| Rate of Length in Clustering(%) | 92.99 |
| Number of Sequence in Ordering | 888 |
| Rate of Number in Ordering(%) | 75.83 |
| Length of Sequence in Ordering | 2,624,910,820 |
| Rate of Length in Ordering(%) | 99.31 |
| Number of Sequence in Trunks | 594 |
| Rate of Number in Trunks(%) | 66.89 |
| Length of Sequence in Trunks | 2,262,292,596 |
| Rate of Length in Trunks(%) | 86.19 |
