# Supplementary material for: The reference genome and transcriptome of the limestone langur, Trachypithecus leucocephalus, reveal expansion of genes related to alkali tolerance
Source: BMC Biol. 2021 Apr 8;19:67. doi: 10.1186/s12915-021-00998-2 (PMC8034193; doi:10.1186/s12915-021-00998-2)
Supplement: Supplementary file 12 — Additional file 12: Table S7. Summary of the functional gene annotation of T. leucocephalus. [file 12915_2021_998_MOESM12_ESM.docx]

| **Additional file 12: Table S*7: Summary of the functional gene annotation of T. leucocephalus.*** | | |
| --- | --- | --- |
| Database | Count | Percentage (%) |
| Total | 20,925 | 100.00 |
| eggNOG | 17,350 | 82.92 |
| NR | 20,092 | 96.02 |
| BLASTP | 19,531 | 93.34 |
| Map | 7,435 | 35.53 |
| GO | 18,836 | 90.02 |
| BLASTX | 19,608 | 93.71 |
| KO | 12,492 | 59.70 |
| PFAM | 15,796 | 75.49 |
| Annotated | 20,131 | 96.21 |
| Unannotated | 794 | 3.80 |
