# Supplementary material for: The reference genome and transcriptome of the limestone langur, Trachypithecus leucocephalus, reveal expansion of genes related to alkali tolerance
Source: BMC Biol. 2021 Apr 8;19:67. doi: 10.1186/s12915-021-00998-2 (PMC8034193; doi:10.1186/s12915-021-00998-2)
Supplement: Supplementary file 13 — Additional file 13: Table S8. Summary of non-coding RNAs for T. leucocephalus. [file 12915_2021_998_MOESM13_ESM.docx]

| **Additional file 13: Table S8: Summary of non-coding RNAs for T. leucocephalus.** | | | | | |
| --- | --- | --- | --- | --- | --- |
|  | Type | Copy | Average length(bp) | Total length(bp) | %of genome |
| miRNA | | 11,113 | 245.3374 | 2726434 | 0.09572 |
| tRNA | | 736 | 122.0598 | 89836 | 0.003154 |
| rRNA | 18S | 22 | 378.2273 | 8321 | 0.000292 |
|  | 28S | 199 | 214.1055 | 42607 | 0.001496 |
|  | 5.8S | 8 | 120.125 | 961 | 0.000034 |
|  | 5S | 507 | 74.84615 | 37947 | 0.001332 |
