# Supplementary material for: The reference genome and transcriptome of the limestone langur, Trachypithecus leucocephalus, reveal expansion of genes related to alkali tolerance
Source: BMC Biol. 2021 Apr 8;19:67. doi: 10.1186/s12915-021-00998-2 (PMC8034193; doi:10.1186/s12915-021-00998-2)
Supplement: Supplementary file 14 — Additional file 14: Table S9. Summary of TE for T. leucocephalus. [file 12915_2021_998_MOESM14_ESM.docx]

| **Additional file 14: Table S9: Summary of TE for T. leucocephalus.** | | | | | |  |  |  |
| --- | --- | --- | --- | --- | --- | --- | --- | --- |
| Type | Repbase TEs | | TE proteins | | RepeatModeler | | Combined TEs | |
|  | Length(bp) | Percentage(%) | Length(bp) | Percentage(%) | Length(bp) | Percentage(%) | Length(bp) | Percentage(%) |
| DNA | 99,370,596 | 3.49 | 6,836,906 | 0.24 | 25,516,484 | 0.9 | 100,148,462 | 3.52 |
| LINE | 629,428,664 | 22.1 | 309,963,282 | 10.88 | 751,693,206 | 26.39 | 964,229,945 | 33.85 |
| SINE | 418,189,903 | 14.68 | 0 | 0 | 0 | 0 | 418,189,903 | 14.68 |
| LTR | 251,327,083 | 8.82 | 42,832,961 | 1.5 | 65,267,518 | 2.29 | 259,510,789 | 9.11 |
| Unknown | 775,353 | 0.03 | 111 | 0 | 366,927,335 | 12.88 | 367,701,828 | 12.91 |
| Other | 54,926,771 | 1.93 | 72,878,795 | 2.56 | 47,581,322 | 1.67 | 92,277,401 | 3.24 |
| Total | 1,453,751,200 | 51.04 | 429,357,045 | 15.07 | 1,191,460,948 | 41.83 | 1,502,678,531 | 52.76 |
