# Supplementary material for: The reference genome and transcriptome of the limestone langur, Trachypithecus leucocephalus, reveal expansion of genes related to alkali tolerance
Source: BMC Biol. 2021 Apr 8;19:67. doi: 10.1186/s12915-021-00998-2 (PMC8034193; doi:10.1186/s12915-021-00998-2)
Supplement: Supplementary file 15 — Additional file 15: Table S10. The detailed information (version, website links, etc.) of genome assemblies of 14 other primates used in this study. [file 12915_2021_998_MOESM15_ESM.docx]

| **Additional file 15: Table S10: The detailed information (version, website links, etc.) of genome assemblies of 14 other primates used in this study.** | | | | |
| --- | --- | --- | --- | --- |
| Family | Genus | Species | Version | Website |
| Cercopithecidae | Colobinae | Trachypithecus francoisi | Tfra_2.0 | https://www.ncbi.nlm.nih.gov/genome/?term=Trachypithecus+francoisi |
| Hylobatidae | Nomascus | Nomascus leucogenys | Nleu_3.0 | https://www.ncbi.nlm.nih.gov/genome/?term=Nomascus_leucogenys |
| homindae | homo | Homo sapiens | GRCh38.p12 | https://www.ncbi.nlm.nih.gov/genome/?term=Homo_sapiens |
| Hominidae | Pan | Pan troglodytes | Clint_PTRv2 | https://www.ncbi.nlm.nih.gov/genome/?term=Pan_troglodytes_verus |
| Hominidae | Pan | Pan paniscus | panpan1.1 | https://www.ncbi.nlm.nih.gov/genome/?term=Pan_paniscus |
| Cercopithecidae | Piliocolobus | Piliocolobus tephrosceles | ASM277652v1 | https://www.ncbi.nlm.nih.gov/genome/?term=Piliocolobus%20tephrosceles |
| Cercopithecidae | Rhinopithecus | Rhinopithecus roxellana | Rrox_v1 | https://www.ncbi.nlm.nih.gov/genome/?term=Rhinopithecus%20roxellana |
| Cercopithecidae | Rhinopithecus | Rhinopithecus bieti | ASM169854v1 | https://www.ncbi.nlm.nih.gov/genome/?term=Rhinopithecus%20bieti |
| Cercopithecidae | Chlorocebus | Chlorocebus sabaeus | Chlorocebus_sabeus_1.1 | https://www.ncbi.nlm.nih.gov/genome/?term=Chlorocebus%20sabaeus |
| Cercopithecidae | Papio | Papio anubis | Panu_3.0 | https://www.ncbi.nlm.nih.gov/genome/?term=Papio%20anubis |
| Cercopithecidae | Macaca | Macaca nemestrina | Mnem_1.0 | https://www.ncbi.nlm.nih.gov/genome/?term=Macaca%20nemestrina |
| Cercopithecidae | Macaca | Macaca mulatta | Mmul_8.0.1 | <https://www.ncbi.nlm.nih.gov/genome/?term=Macaca%20mulatta> |
| Cercopithecidae | Macaca | Macaca fascicularis | Macaca_fascicularis_5.0 | https://www.ncbi.nlm.nih.gov/genome/?term=Macaca%20fascicularis |
| Cercopithecidae | Cercocebus | Cercocebus atys | Caty_1.0 | https://www.ncbi.nlm.nih.gov/genome/?term=Cercocebus%20atys |
| Cercopithecidae | Colobus | Colobus angolensis | Cang.pa_1.0 | https://www.ncbi.nlm.nih.gov/genome/?term=Colobus%20angolensis |
