# Supplementary material for: The reference genome and transcriptome of the limestone langur, Trachypithecus leucocephalus, reveal expansion of genes related to alkali tolerance
Source: BMC Biol. 2021 Apr 8;19:67. doi: 10.1186/s12915-021-00998-2 (PMC8034193; doi:10.1186/s12915-021-00998-2)
Supplement: Supplementary file 16 — Additional file 16: Table S11. Summary of the repeat TE classes for T. leucocephalus. [file 12915_2021_998_MOESM16_ESM.docx]

| **Additional file 16: Table S11: Summary of the repeat TE classes for T. leucocephalus.** | | | | | |
| --- | --- | --- | --- | --- | --- |
| Species | Total genes | Unclustered genes | Families | Unique families | Ave. genes per family |
| *T.leucocephalus* | 20,925 | 2,772 | 13,194 | 129 | 1.38 |
| *C.atys* | 19,682 | 413 | 17,095 | 3 | 1.13 |
| *C.sabaeus* | 20,091 | 912 | 17,112 | 9 | 1.12 |
| *C.angolensis* | 18,906 | 240 | 16,646 | 0 | 1.12 |
| *H.sapiens* | 22,605 | 270 | 17,408 | 38 | 1.28 |
| *M.fascicularis* | 19,920 | 268 | 17,321 | 0 | 1.13 |
| *M.mulatta* | 19,968 | 264 | 17,234 | 5 | 1.14 |
| *M.nemestrina* | 20,192 | 463 | 17,453 | 2 | 1.13 |
| *N.leucogenys* | 18,885 | 728 | 17,886 | 54 | 1.02 |
| *P.paniscus* | 19,694 | 295 | 17,410 | 0 | 1.11 |
| *P.troglodytes* | 20,939 | 472 | 17,853 | 13 | 1.15 |
| *P.anubis* | 20,267 | 379 | 17,378 | 3 | 1.14 |
| *P.tephrosceles* | 20,683 | 845 | 17,255 | 36 | 1.15 |
| *R.bieti* | 19,564 | 552 | 16,743 | 2 | 1.14 |
| *R.roxellana* | 19,546 | 298 | 16,977 | 2 | 1.13 |
