# Supplementary material for: The reference genome and transcriptome of the limestone langur, Trachypithecus leucocephalus, reveal expansion of genes related to alkali tolerance
Source: BMC Biol. 2021 Apr 8;19:67. doi: 10.1186/s12915-021-00998-2 (PMC8034193; doi:10.1186/s12915-021-00998-2)
Supplement: Supplementary file 17 — Additional file 17: Table S12. Top 10 enrichment pathways of the special gene families of T. leucocephalus. [file 12915_2021_998_MOESM17_ESM.docx]

| **Additional file 17: Table S12: Top 10 enrichment pathways of the special gene families of T. leucocephalus.** | | | | | | | | |
| --- | --- | --- | --- | --- | --- | --- | --- | --- |
| Name | Map | Count1 | Count2 | Count3 | Count4 | p-value | q-value | Genes in background |
| Ether lipid metabolism | map00565 | 4 | 22 | 71 | 7511 | 0.000111 | 0.03735 | evm.model.utg134.13\|K01047;evm.model.utg209.14\|K16860;evm.model.utg221.19\|K01019;evm.model.utg221.22\|K01019;evm.model.utg2594.7\|K01047;evm.model.utg265.23\|K16795;evm.model.utg299.58\|K01115;evm.model.utg33.78\|K00993;evm.model.utg331.44\|K13644;evm.model.utg367.60\|K16817;evm.model.utg415.7\|K04628;evm.model.utg435.59\|K16342;evm.model.utg440.32\|K01062;evm.model.utg5537.13\|K01080;evm.model.utg646.37\|K01047;evm.model.utg646.39\|K01047;evm.model.utg646.40\|K01047;evm.model.utg78.30\|K13510;evm.model.utg914.4\|K16342;evm.model.utg914.7\|K16342;evm.model.utg914.8\|K16342;evm.model.utg914.9\|K16342;evm.model.utg95.29\|K01080;evm.model.utg97.190\|K16795;evm.model.utg994.10\|K13512;evm.model.utg994.11\|K13512 |
| Inflammatory mediator regulation of TRP channels | map04750 | 6 | 91 | 69 | 7442 | 0.000364 | 0.041049 | evm.model.utg1097.1\|K04440;evm.model.utg118.26\|K04259;evm.model.utg1186.80\|K02582;evm.model.utg1259.7\|K04432;evm.model.utg128.1\|K05459;evm.model.utg1335.18\|K19663;evm.model.utg136.2\|K06068;evm.model.utg145.120\|K08045;evm.model.utg151.13\|K04440;evm.model.utg154.55\|K04830;evm.model.utg1595.50\|K02183;evm.model.utg184.129\|K04269;evm.model.utg19.57\|K04261;evm.model.utg195.74\|K08049;evm.model.utg203.135\|K08042;evm.model.utg203.136\|K08042;evm.model.utg203.140\|K08042;evm.model.utg2051.1\|K00458;evm.model.utg206.44\|K05222;evm.model.utg236.33\|K04832;evm.model.utg242.31\|K05858;evm.model.utg243.79\|K00922;evm.model.utg246.52\|K02649;evm.model.utg2623.4\|K00922;evm.model.utg263.36\|K04515;evm.model.utg272.59\|K00458;evm.model.utg2758.13\|K02677;evm.model.utg2758.9\|K02677;evm.model.utg276.1\|K08044;evm.model.utg284.22\|K18050;evm.model.utg284.27\|K18050;evm.model.utg296.4\|K04234;evm.model.utg319.7\|K02183;evm.model.utg319.9\|K02183;evm.model.utg324.14\|K19662;evm.model.utg324.16\|K19662;evm.model.utg324.17\|K19662;evm.model.utg3241.1\|K04157;evm.model.utg33.150\|K08043;evm.model.utg33.151\|K08043;evm.model.utg33.22\|K06269;evm.model.utg367.41\|K05858;evm.model.utg377.20\|K07418;evm.model.utg383.1\|K04433;evm.model.utg384.80\|K04157;evm.model.utg39.131\|K08048;evm.model.utg39.132\|K08048;evm.model.utg39.134\|K08048;evm.model.utg39.135\|K08048;evm.model.utg392.18\|K04723;evm.model.utg392.19\|K04723;evm.model.utg414.54\|K04958;evm.model.utg423.31\|K04632;evm.model.utg423.35\|K04632;evm.model.utg435.59\|K16342;evm.model.utg441.104\|K06269;evm.model.utg441.84\|K04973;evm.model.utg451.24\|K02183;evm.model.utg46.117\|K04515;evm.model.utg461.100\|K04829;evm.model.utg461.140\|K08046;evm.model.utg462.118\|K05859;evm.model.utg47.70\|K08041;evm.model.utg47.71\|K08041;evm.model.utg47.72\|K08041;evm.model.utg47.93\|K04515;evm.model.utg483.42\|K04828;evm.model.utg483.50\|K04828;evm.model.utg495.117\|K01116;evm.model.utg495.71\|K05704;evm.model.utg525.7\|K00922;evm.model.utg64.92\|K04984;evm.model.utg64.93\|K04984;evm.model.utg642.25\|K04441;evm.model.utg643.59\|K02649;evm.model.utg643.60\|K02649;evm.model.utg653.2\|K04971;evm.model.utg653.3\|K04971;evm.model.utg661.76\|K06269;evm.model.utg67.168\|K04634;evm.model.utg67.169\|K04634;evm.model.utg67.170\|K04634;evm.model.utg67.171\|K04634;evm.model.utg67.84\|K04345;evm.model.utg76.34\|K04831;evm.model.utg833.26\|K04634;evm.model.utg833.27\|K04634;evm.model.utg853.56\|K18051;evm.model.utg90.30\|K03176;evm.model.utg900.1\|K04959;evm.model.utg900.3\|K04959;evm.model.utg914.4\|K16342;evm.model.utg914.7\|K16342;evm.model.utg914.8\|K16342;evm.model.utg914.9\|K16342;evm.model.utg949.9\|K04971;evm.model.utg95.56\|K07418 |
| MAPK signaling pathway | map04010 | 10 | 259 | 65 | 7274 | 0.000281 | 0.041049 | evm.model.utg10.66\|K02187;evm.model.utg101.14\|K04369;evm.model.utg1034.20\|K12363;evm.model.utg1076.19\|K02833;evm.model.utg1076.55\|K04459;evm.model.utg1076.71\|K13769;evm.model.utg10900.1\|K09253;evm.model.utg1097.1\|K04440;evm.model.utg11.136\|K04437;evm.model.utg11.229\|K04443;evm.model.utg11.230\|K04443;evm.model.utg11.254\|K04376;evm.model.utg11.84\|K04735;evm.model.utg11116.2\|K04430;evm.model.utg114.22\|K04363;evm.model.utg114.26\|K05098;evm.model.utg1164.5\|K04377;evm.model.utg1186.80\|K02582;evm.model.utg1187.19\|K04405;evm.model.utg1223.11\|K05462;evm.model.utg123.30\|K04393;evm.model.utg1239.37\|K04344;evm.model.utg1259.7\|K04432;evm.model.utg127.1\|K04410;evm.model.utg128.1\|K05459;evm.model.utg133.144\|K04437;evm.model.utg1335.18\|K19663;evm.model.utg134.16\|K04393;evm.model.utg134.8\|K04357;evm.model.utg136.10\|K04851;evm.model.utg136.18\|K04860;evm.model.utg136.19\|K04860;evm.model.utg136.22\|K04860;evm.model.utg136.24\|K04860;evm.model.utg136.25\|K04860;evm.model.utg136.28\|K04860;evm.model.utg1365.9\|K04468;evm.model.utg140.57\|K04378;evm.model.utg142.34\|K03283;evm.model.utg142.35\|K03283;evm.model.utg143.132\|K04445;evm.model.utg143.135\|K04445;evm.model.utg1435.4\|K04466;evm.model.utg147.133\|K04729;evm.model.utg151.13\|K04440;evm.model.utg1513.7\|K04358;evm.model.utg155.40\|K04426;evm.model.utg155.41\|K04426;evm.model.utg15666.1\|K08052;evm.model.utg1594.21\|K04358;evm.model.utg16.32\|K07860;evm.model.utg161.42\|K04358;evm.model.utg1651.30\|K04371;evm.model.utg1651.41\|K04429;evm.model.utg168.2\|K04402;evm.model.utg169.103\|K04429;evm.model.utg1692.2\|K05450;evm.model.utg177.31\|K04454;evm.model.utg177.42\|K04352;evm.model.utg177.43\|K04352;evm.model.utg1802.9\|K16859;evm.model.utg19.6\|K04372;evm.model.utg1901.2\|K07836;evm.model.utg193.66\|K04355;evm.model.utg201.33\|K04393;evm.model.utg202.14\|K04358;evm.model.utg205.41\|K04733;evm.model.utg2051.31\|K04358;evm.model.utg209.8\|K04456;evm.model.utg21.54\|K04348;evm.model.utg21.63\|K04370;evm.model.utg222.109\|K05866;evm.model.utg2247.3\|K03283;evm.model.utg226.35\|K03099;evm.model.utg234.54\|K13377;evm.model.utg234.68\|K04379;evm.model.utg236.25\|K05450;evm.model.utg236.26\|K05450;evm.model.utg2396.1\|K08052;evm.model.utg242.56\|K04350;evm.model.utg2501.2\|K05449;evm.model.utg2501.3\|K05449;evm.model.utg252.16\|K04459;evm.model.utg254.1\|K04392;evm.model.utg257.9\|K04865;evm.model.utg263.114\|K18496;evm.model.utg263.186\|K04391;evm.model.utg263.39\|K05089;evm.model.utg263.40\|K05090;evm.model.utg272.7\|K04439;evm.model.utg274.21\|K05087;evm.model.utg274.25\|K05087;evm.model.utg2758.13\|K02677;evm.model.utg2758.3\|K04866;evm.model.utg2758.5\|K04869;evm.model.utg2758.6\|K04870;evm.model.utg2758.9\|K02677;evm.model.utg2807.3\|K04437;evm.model.utg2807.4\|K04437;evm.model.utg282.22\|K03283;evm.model.utg283.37\|K06268;evm.model.utg284.44\|K04461;evm.model.utg287.6\|K04457;evm.model.utg2879.6\|K18018;evm.model.utg2879.7\|K18018;evm.model.utg2891.26\|K04438;evm.model.utg290.53\|K17614;evm.model.utg2902.1\|K04375;evm.model.utg291.18\|K04366;evm.model.utg299.18\|K04462;evm.model.utg3116.1\|K04372;evm.model.utg3116.14\|K04402;evm.model.utg324.14\|K19662;evm.model.utg324.16\|K19662;evm.model.utg324.17\|K19662;evm.model.utg324.19\|K04868;evm.model.utg326.82\|K04358;evm.model.utg326.96\|K04469;evm.model.utg331.29\|K04353;evm.model.utg331.68\|K05453;evm.model.utg332.5\|K04393;evm.model.utg346.110\|K05121;evm.model.utg346.24\|K07836;evm.model.utg35.136\|K03171;evm.model.utg35.91\|K17333;evm.model.utg350.38\|K08053;evm.model.utg354.129\|K04424;evm.model.utg354.95\|K04450;evm.model.utg357.10\|K04371;evm.model.utg359.19\|K04458;evm.model.utg363.40\|K05449;evm.model.utg363.41\|K05449;evm.model.utg367.26\|K04414;evm.model.utg367.32\|K16510;evm.model.utg3690.3\|K08845;evm.model.utg3690.4\|K08845;evm.model.utg375.81\|K04420;evm.model.utg377.57\|K04422;evm.model.utg379.3\|K04358;evm.model.utg379.4\|K04358;evm.model.utg383.1\|K04433;evm.model.utg3878.10\|K17614;evm.model.utg392.10\|K04358;evm.model.utg392.18\|K04723;evm.model.utg392.19\|K04723;evm.model.utg3925.6\|K07829;evm.model.utg394.36\|K04859;evm.model.utg394.42\|K04444;evm.model.utg406.4\|K04358;evm.model.utg407.35\|K04358;evm.model.utg407.37\|K04358;evm.model.utg407.69\|K04850;evm.model.utg407.72\|K04850;evm.model.utg407.73\|K04850;evm.model.utg407.82\|K04850;evm.model.utg407.87\|K04861;evm.model.utg416.79\|K05092;evm.model.utg417.16\|K04409;evm.model.utg422.41\|K04421;evm.model.utg426.22\|K03283;evm.model.utg426.23\|K03283;evm.model.utg426.34\|K05462;evm.model.utg428.14\|K04459;evm.model.utg428.6\|K03283;evm.model.utg433.16\|K04459;evm.model.utg435.30\|K04358;evm.model.utg435.59\|K16342;evm.model.utg438.15\|K07831;evm.model.utg438.16\|K07831;evm.model.utg439.6\|K05466;evm.model.utg440.30\|K04381;evm.model.utg451.30\|K04463;evm.model.utg46.215\|K04415;evm.model.utg461.138\|K04864;evm.model.utg461.61\|K04465;evm.model.utg467.5\|K04423;evm.model.utg479.2\|K04373;evm.model.utg479.24\|K04425;evm.model.utg483.12\|K08052;evm.model.utg483.8\|K08052;evm.model.utg483.9\|K08052;evm.model.utg490.26\|K13375;evm.model.utg501.7\|K04431;evm.model.utg506.29\|K21278;evm.model.utg516.9\|K07209;evm.model.utg524.60\|K04852;evm.model.utg524.61\|K04852;evm.model.utg524.62\|K04852;evm.model.utg524.64\|K04852;evm.model.utg527.11\|K04361;evm.model.utg527.2\|K04393;evm.model.utg539.82\|K04459;evm.model.utg541.3\|K04416;evm.model.utg5481.3\|K07210;evm.model.utg5481.4\|K04374;evm.model.utg597.27\|K04412;evm.model.utg597.28\|K04412;evm.model.utg598.12\|K07830;evm.model.utg599.65\|K02583;evm.model.utg599.91\|K04854;evm.model.utg6191.1\|K04358;evm.model.utg62.13\|K04392;evm.model.utg624.3\|K12326;evm.model.utg624.4\|K12326;evm.model.utg63.16\|K04434;evm.model.utg642.25\|K04441;evm.model.utg643.51\|K04449;evm.model.utg655.11\|K04360;evm.model.utg655.13\|K04360;evm.model.utg655.8\|K04360;evm.model.utg655.9\|K04360;evm.model.utg66.2\|K07827;evm.model.utg661.150\|K04735;evm.model.utg661.153\|K04419;evm.model.utg661.21\|K04358;evm.model.utg661.22\|K04358;evm.model.utg661.24\|K04358;evm.model.utg67.84\|K04345;evm.model.utg672.6\|K04393;evm.model.utg683.27\|K04346;evm.model.utg688.18\|K04362;evm.model.utg69.2\|K04858;evm.model.utg69.24\|K03283;evm.model.utg694.17\|K05094;evm.model.utg700.18\|K03283;evm.model.utg700.6\|K04453;evm.model.utg712.15\|K04455;evm.model.utg73.33\|K04459;evm.model.utg74.102\|K04428;evm.model.utg74.104\|K04428;evm.model.utg74.169\|K04373;evm.model.utg74.170\|K04373;evm.model.utg797.61\|K04856;evm.model.utg797.62\|K04856;evm.model.utg797.64\|K04374;evm.model.utg797.68\|K04403;evm.model.utg797.72\|K17386;evm.model.utg8.93\|K02187;evm.model.utg84.108\|K03156;evm.model.utg84.84\|K03283;evm.model.utg84.86\|K03283;evm.model.utg85.21\|K04452;evm.model.utg853.40\|K04457;evm.model.utg861.4\|K18498;evm.model.utg875.1\|K09253;evm.model.utg875.56\|K04460;evm.model.utg892.38\|K03173;evm.model.utg892.80\|K04849;evm.model.utg9.14\|K03175;evm.model.utg90.30\|K03176;evm.model.utg90.89\|K05462;evm.model.utg912.37\|K07836;evm.model.utg914.4\|K16342;evm.model.utg914.7\|K16342;evm.model.utg914.8\|K16342;evm.model.utg914.9\|K16342;evm.model.utg915.31\|K04366;evm.model.utg92.39\|K20216;evm.model.utg92.8\|K13376;evm.model.utg94.15\|K04862;evm.model.utg95.133\|K04402;evm.model.utg95.45\|K04448;evm.model.utg95.46\|K04448;evm.model.utg95.9\|K04456;evm.model.utg967.64\|K04855;evm.model.utg97.115\|K05461;evm.model.utg97.116\|K04459;evm.model.utg97.117\|K04459;evm.model.utg979.9\|K04435 |
| Long-term depression | map04730 | 5 | 69 | 70 | 7464 | 0.000772 | 0.065235 | evm.model.utg101.14\|K04369;evm.model.utg1034.18\|K04961;evm.model.utg1076.19\|K02833;evm.model.utg1239.37\|K04344;evm.model.utg128.1\|K05459;evm.model.utg1335.18\|K19663;evm.model.utg1385.4\|K04635;evm.model.utg156.83\|K05256;evm.model.utg165.5\|K04603;evm.model.utg165.6\|K04603;evm.model.utg165.9\|K04603;evm.model.utg1651.30\|K04371;evm.model.utg1704.1\|K05199;evm.model.utg1704.2\|K05199;evm.model.utg1955.6\|K04639;evm.model.utg20.17\|K07376;evm.model.utg20.18\|K07376;evm.model.utg20.19\|K07376;evm.model.utg20.24\|K07376;evm.model.utg20.27\|K07376;evm.model.utg236.23\|K05198;evm.model.utg236.35\|K12318;evm.model.utg242.31\|K05858;evm.model.utg274.21\|K05087;evm.model.utg274.25\|K05087;evm.model.utg2758.13\|K02677;evm.model.utg2758.9\|K02677;evm.model.utg291.18\|K04366;evm.model.utg324.14\|K19662;evm.model.utg324.16\|K19662;evm.model.utg324.17\|K19662;evm.model.utg341.3\|K05207;evm.model.utg341.4\|K05207;evm.model.utg357.10\|K04371;evm.model.utg367.41\|K05858;evm.model.utg3690.3\|K08845;evm.model.utg3690.4\|K08845;evm.model.utg37.10\|K12318;evm.model.utg37.8\|K12318;evm.model.utg37.9\|K12318;evm.model.utg375.35\|K04382;evm.model.utg389.1\|K05207;evm.model.utg389.2\|K05207;evm.model.utg389.3\|K05207;evm.model.utg394.24\|K04630;evm.model.utg397.40\|K04635;evm.model.utg397.41\|K04635;evm.model.utg414.54\|K04958;evm.model.utg423.31\|K04632;evm.model.utg423.35\|K04632;evm.model.utg424.24\|K05197;evm.model.utg435.59\|K16342;evm.model.utg473.6\|K04630;evm.model.utg5080.1\|K05199;evm.model.utg623.21\|K08067;evm.model.utg66.2\|K07827;evm.model.utg67.168\|K04634;evm.model.utg67.169\|K04634;evm.model.utg67.170\|K04634;evm.model.utg67.171\|K04634;evm.model.utg683.27\|K04346;evm.model.utg721.31\|K04382;evm.model.utg78.37\|K04534;evm.model.utg78.39\|K04534;evm.model.utg833.26\|K04634;evm.model.utg833.27\|K04634;evm.model.utg900.1\|K04959;evm.model.utg900.3\|K04959;evm.model.utg914.4\|K16342;evm.model.utg914.7\|K16342;evm.model.utg914.8\|K16342;evm.model.utg914.9\|K16342;evm.model.utg915.31\|K04366;evm.model.utg965.15\|K04535 |
| Arginine biosynthesis | map00220 | 4 | 45 | 71 | 7488 | 0.00132 | 0.088577 | evm.model.utg1012.6\|K13241;evm.model.utg1085.2\|K01940;evm.model.utg11.13\|K01940;evm.model.utg11.287\|K01940;evm.model.utg1139.13\|K14455;evm.model.utg1139.14\|K14455;evm.model.utg1365.2\|K13241;evm.model.utg1365.6\|K13241;evm.model.utg141.33\|K01476;evm.model.utg1435.43\|K11067;evm.model.utg154.52\|K13242;evm.model.utg1570.10\|K01940;evm.model.utg1588.2\|K00261;evm.model.utg220.12\|K14455;evm.model.utg2275.3\|K13241;evm.model.utg2597.3\|K13241;evm.model.utg263.93\|K01940;evm.model.utg263.94\|K01940;evm.model.utg278.12\|K14455;evm.model.utg278.13\|K14455;evm.model.utg2859.1\|K01940;evm.model.utg2859.2\|K01940;evm.model.utg2859.3\|K01940;evm.model.utg299.99\|K01940;evm.model.utg326.30\|K14454;evm.model.utg349.27\|K01940;evm.model.utg37.12\|K01940;evm.model.utg37.13\|K01940;evm.model.utg395.67\|K01940;evm.model.utg40.79\|K01940;evm.model.utg47.154\|K01940;evm.model.utg524.70\|K01915;evm.model.utg56.20\|K00261;evm.model.utg5672.2\|K00261;evm.model.utg584.12\|K01425;evm.model.utg598.65\|K01915;evm.model.utg600.70\|K00814;evm.model.utg67.173\|K01940;evm.model.utg73.35\|K14677;evm.model.utg73.36\|K14677;evm.model.utg745.36\|K14455;evm.model.utg7745.1\|K00611;evm.model.utg7745.2\|K00611;evm.model.utg775.10\|K01940;evm.model.utg775.11\|K01940;evm.model.utg78.92\|K14455;evm.model.utg809.2\|K13241;evm.model.utg822.11\|K00261;evm.model.utg866.35\|K01915 |
| MicroRNAs in cancer | map05206 | 6 | 122 | 69 | 7411 | 0.001572 | 0.088577 | evm.model.utg10.66\|K02187;evm.model.utg101.14\|K04369;evm.model.utg1052.24\|K07203;evm.model.utg1076.19\|K02833;evm.model.utg11.412\|K00510;evm.model.utg11.413\|K00510;evm.model.utg1118.8\|K00312;evm.model.utg114.22\|K04363;evm.model.utg1164.5\|K04377;evm.model.utg1223.17\|K02085;evm.model.utg123.37\|K09299;evm.model.utg124.9\|K05762;evm.model.utg125.12\|K06643;evm.model.utg125.45\|K08007;evm.model.utg1311.37\|K17449;evm.model.utg1335.18\|K19663;evm.model.utg13506.2\|K05762;evm.model.utg1379.1\|K00510;evm.model.utg143.132\|K04445;evm.model.utg143.135\|K04445;evm.model.utg146.1\|K04702;evm.model.utg148.17\|K11411;evm.model.utg149.14\|K17383;evm.model.utg154.5\|K11430;evm.model.utg162.23\|K12886;evm.model.utg1866.28\|K02539;evm.model.utg189.24\|K12886;evm.model.utg189.25\|K12886;evm.model.utg190.56\|K02091;evm.model.utg190.57\|K02091;evm.model.utg190.59\|K02091;evm.model.utg1921.1\|K14021;evm.model.utg195.63\|K00510;evm.model.utg1980.12\|K07204;evm.model.utg1980.8\|K07204;evm.model.utg1980.9\|K07204;evm.model.utg199.65\|K04513;evm.model.utg201.11\|K09389;evm.model.utg2028.3\|K17390;evm.model.utg213.35\|K10145;evm.model.utg220.25\|K10139;evm.model.utg220.28\|K02161;evm.model.utg222.109\|K05866;evm.model.utg226.35\|K03099;evm.model.utg242.35\|K17460;evm.model.utg242.44\|K16857;evm.model.utg25.78\|K05665;evm.model.utg252.9\|K16865;evm.model.utg259.19\|K07436;evm.model.utg263.39\|K05089;evm.model.utg2758.13\|K02677;evm.model.utg2758.9\|K02677;evm.model.utg276.36\|K02163;evm.model.utg284.22\|K18050;evm.model.utg284.27\|K18050;evm.model.utg2891.26\|K04438;evm.model.utg291.18\|K04366;evm.model.utg303.19\|K10373;evm.model.utg324.14\|K19662;evm.model.utg324.16\|K19662;evm.model.utg324.17\|K19662;evm.model.utg338.19\|K07410;evm.model.utg346.81\|K06621;evm.model.utg354.79\|K09295;evm.model.utg359.42\|K06643;evm.model.utg367.16\|K17390;evm.model.utg37.26\|K04728;evm.model.utg378.6\|K10373;evm.model.utg391.10\|K06645;evm.model.utg391.48\|K04513;evm.model.utg392.30\|K10149;evm.model.utg392.31\|K10149;evm.model.utg392.32\|K10149;evm.model.utg394.31\|K09850;evm.model.utg407.40\|K10151;evm.model.utg41.23\|K12035;evm.model.utg41.24\|K12035;evm.model.utg421.27\|K04694;evm.model.utg422.6\|K00312;evm.model.utg422.7\|K00312;evm.model.utg422.8\|K00312;evm.model.utg428.22\|K02329;evm.model.utg433.19\|K06624;evm.model.utg440.30\|K04381;evm.model.utg46.151\|K08270;evm.model.utg462.118\|K05859;evm.model.utg486.36\|K11987;evm.model.utg495.117\|K01116;evm.model.utg495.16\|K17454;evm.model.utg495.31\|K17399;evm.model.utg498.55\|K06626;evm.model.utg516.9\|K07209;evm.model.utg52.74\|K14021;evm.model.utg52.83\|K08670;evm.model.utg520.22\|K17398;evm.model.utg527.11\|K04361;evm.model.utg584.12\|K01425;evm.model.utg590.87\|K01403;evm.model.utg599.51\|K17391;evm.model.utg599.53\|K17391;evm.model.utg642.11\|K06625;evm.model.utg66.2\|K07827;evm.model.utg661.27\|K04503;evm.model.utg694.17\|K05094;evm.model.utg697.2\|K13863;evm.model.utg723.32\|K05867;evm.model.utg74.58\|K12561;evm.model.utg745.63\|K12886;evm.model.utg745.64\|K12886;evm.model.utg768.1\|K17442;evm.model.utg768.5\|K17442;evm.model.utg797.38\|K04498;evm.model.utg797.72\|K17386;evm.model.utg8.93\|K02187;evm.model.utg807.26\|K12886;evm.model.utg834.51\|K07187;evm.model.utg84.24\|K14021;evm.model.utg84.70\|K06252;evm.model.utg84.71\|K06252;evm.model.utg852.24\|K12886;evm.model.utg852.25\|K12886;evm.model.utg892.24\|K02599;evm.model.utg90.96\|K06279;evm.model.utg915.31\|K04366;evm.model.utg92.8\|K13376;evm.model.utg921.41\|K04692;evm.model.utg921.5\|K10605;evm.model.utg94.33\|K05083 |
| Natural killer cell mediated cytotoxicity | map04650 | 5 | 87 | 70 | 7446 | 0.002054 | 0.099203 | evm.model.utg10.66\|K02187;evm.model.utg101.14\|K04369;evm.model.utg1076.19\|K02833;evm.model.utg1140.2\|K07990;evm.model.utg125.38\|K07293;evm.model.utg125.39\|K07293;evm.model.utg1311.37\|K17449;evm.model.utg1335.18\|K19663;evm.model.utg155.30\|K07293;evm.model.utg155.35\|K05132;evm.model.utg156.28\|K07293;evm.model.utg156.29\|K07293;evm.model.utg1595.11\|K05697;evm.model.utg16.32\|K07860;evm.model.utg161.20\|K07293;evm.model.utg161.21\|K07293;evm.model.utg165.48\|K07987;evm.model.utg165.53\|K07986;evm.model.utg165.61\|K07986;evm.model.utg165.63\|K07986;evm.model.utg1651.30\|K04371;evm.model.utg1651.62\|K05718;evm.model.utg168.17\|K05855;evm.model.utg1783.10\|K06464;evm.model.utg1783.9\|K06464;evm.model.utg186.30\|K05697;evm.model.utg21.54\|K04348;evm.model.utg2187.1\|K07361;evm.model.utg226.35\|K03099;evm.model.utg243.79\|K00922;evm.model.utg246.52\|K02649;evm.model.utg254.1\|K04392;evm.model.utg2623.4\|K00922;evm.model.utg2697.2\|K04726;evm.model.utg270.14\|K05730;evm.model.utg270.15\|K05730;evm.model.utg270.17\|K05730;evm.model.utg273.2\|K07293;evm.model.utg273.3\|K07293;evm.model.utg2758.13\|K02677;evm.model.utg2758.9\|K02677;evm.model.utg283.37\|K06268;evm.model.utg291.18\|K04366;evm.model.utg299.68\|K04721;evm.model.utg309.106\|K05131;evm.model.utg324.14\|K19662;evm.model.utg324.16\|K19662;evm.model.utg324.17\|K19662;evm.model.utg346.58\|K05414;evm.model.utg346.59\|K05414;evm.model.utg346.60\|K05414;evm.model.utg346.61\|K05414;evm.model.utg346.62\|K05414;evm.model.utg346.63\|K05414;evm.model.utg346.65\|K05414;evm.model.utg346.67\|K05414;evm.model.utg346.68\|K05414;evm.model.utg346.69\|K05414;evm.model.utg346.70\|K05414;evm.model.utg346.72\|K05414;evm.model.utg357.10\|K04371;evm.model.utg3690.3\|K08845;evm.model.utg3690.4\|K08845;evm.model.utg40.1\|K05730;evm.model.utg40.2\|K05730;evm.model.utg417.16\|K04409;evm.model.utg428.42\|K05871;evm.model.utg462.118\|K05859;evm.model.utg493.46\|K06479;evm.model.utg493.50\|K06582;evm.model.utg495.117\|K01116;evm.model.utg524.85\|K06741;evm.model.utg525.7\|K00922;evm.model.utg564.28\|K04722;evm.model.utg5770.3\|K07981;evm.model.utg62.13\|K04392;evm.model.utg643.59\|K02649;evm.model.utg643.60\|K02649;evm.model.utg66.2\|K07827;evm.model.utg660.12\|K07293;evm.model.utg7446.4\|K05730;evm.model.utg7446.6\|K05730;evm.model.utg772.124\|K06741;evm.model.utg772.127\|K07981;evm.model.utg8.93\|K02187;evm.model.utg84.108\|K03156;evm.model.utg860.60\|K17448;evm.model.utg860.62\|K17448;evm.model.utg868.1\|K04722;evm.model.utg868.2\|K04722;evm.model.utg90.96\|K06279;evm.model.utg915.31\|K04366 |
| Fc gamma R-mediated phagocytosis | map04666 | 5 | 95 | 70 | 7438 | 0.00296 | 0.125039 | evm.model.utg1052.16\|K05765;evm.model.utg1070.30\|K04718;evm.model.utg11.387\|K06478;evm.model.utg113.19\|K00889;evm.model.utg123.30\|K04393;evm.model.utg1232.3\|K00889;evm.model.utg1232.4\|K00889;evm.model.utg1335.18\|K19663;evm.model.utg134.16\|K04393;evm.model.utg136.2\|K06068;evm.model.utg1445.4\|K06274;evm.model.utg16.32\|K07860;evm.model.utg1651.30\|K04371;evm.model.utg168.17\|K05855;evm.model.utg1683.10\|K05765;evm.model.utg17472.3\|K06498;evm.model.utg184.113\|K15909;evm.model.utg184.29\|K12562;evm.model.utg185.31\|K05765;evm.model.utg1866.51\|K00889;evm.model.utg191.19\|K05757;evm.model.utg191.20\|K05757;evm.model.utg20.36\|K05765;evm.model.utg201.12\|K12488;evm.model.utg201.33\|K04393;evm.model.utg209.8\|K04456;evm.model.utg212.3\|K04688;evm.model.utg216.5\|K05765;evm.model.utg2187.5\|K12367;evm.model.utg2187.8\|K12367;evm.model.utg2187.9\|K12367;evm.model.utg22.87\|K13536;evm.model.utg226.32\|K07941;evm.model.utg243.79\|K00922;evm.model.utg246.52\|K02649;evm.model.utg254.1\|K04392;evm.model.utg2594.4\|K05744;evm.model.utg2623.4\|K00922;evm.model.utg270.14\|K05730;evm.model.utg270.15\|K05730;evm.model.utg270.17\|K05730;evm.model.utg2716.7\|K12488;evm.model.utg2716.8\|K12488;evm.model.utg2758.13\|K02677;evm.model.utg2758.9\|K02677;evm.model.utg282.23\|K06472;evm.model.utg284.22\|K18050;evm.model.utg284.27\|K18050;evm.model.utg2891.26\|K04438;evm.model.utg291.18\|K04366;evm.model.utg299.58\|K01115;evm.model.utg310.25\|K05768;evm.model.utg324.14\|K19662;evm.model.utg324.16\|K19662;evm.model.utg324.17\|K19662;evm.model.utg332.5\|K04393;evm.model.utg357.10\|K04371;evm.model.utg384.131\|K03084;evm.model.utg395.155\|K05756;evm.model.utg397.2\|K08011;evm.model.utg397.59\|K05743;evm.model.utg399.2\|K05757;evm.model.utg399.3\|K05757;evm.model.utg40.1\|K05730;evm.model.utg40.2\|K05730;evm.model.utg4114.2\|K05744;evm.model.utg417.16\|K04409;evm.model.utg435.38\|K04718;evm.model.utg447.18\|K05765;evm.model.utg462.118\|K05859;evm.model.utg47.161\|K12562;evm.model.utg47.163\|K12562;evm.model.utg479.27\|K05748;evm.model.utg495.117\|K01116;evm.model.utg495.43\|K08893;evm.model.utg512.11\|K05754;evm.model.utg524.96\|K05754;evm.model.utg525.7\|K00922;evm.model.utg527.2\|K04393;evm.model.utg5537.13\|K01080;evm.model.utg58.71\|K05756;evm.model.utg588.37\|K05756;evm.model.utg62.13\|K04392;evm.model.utg643.59\|K02649;evm.model.utg643.60\|K02649;evm.model.utg645.3\|K05757;evm.model.utg645.4\|K05757;evm.model.utg661.141\|K05765;evm.model.utg661.73\|K04688;evm.model.utg672.6\|K04393;evm.model.utg74.58\|K12561;evm.model.utg7446.4\|K05730;evm.model.utg7446.6\|K05730;evm.model.utg875.21\|K06274;evm.model.utg88.72\|K12559;evm.model.utg88.73\|K12559;evm.model.utg915.31\|K04366;evm.model.utg95.29\|K01080;evm.model.utg95.9\|K04456;evm.model.utg95.98\|K05765 |
| Proteoglycans in cancer | map05205 | 7 | 207 | 68 | 7326 | 0.004957 | 0.186166 | evm.model.utg10.66\|K02187;evm.model.utg101.14\|K04369;evm.model.utg1019.3\|K03258;evm.model.utg1052.24\|K07203;evm.model.utg10697.1\|K10164;evm.model.utg1076.19\|K02833;evm.model.utg1076.71\|K13769;evm.model.utg11.136\|K04437;evm.model.utg11.147\|K06226;evm.model.utg11.331\|K12329;evm.model.utg11.42\|K12958;evm.model.utg11.43\|K06278;evm.model.utg11.52\|K00182;evm.model.utg11.53\|K00182;evm.model.utg11.69\|K01558;evm.model.utg1118.7\|K01064;evm.model.utg1118.8\|K00312;evm.model.utg114.26\|K05098;evm.model.utg1164.5\|K04377;evm.model.utg123.30\|K04393;evm.model.utg124.31\|K09543;evm.model.utg124.9\|K05762;evm.model.utg125.12\|K06643;evm.model.utg125.38\|K07293;evm.model.utg125.39\|K07293;evm.model.utg125.41\|K04707;evm.model.utg125.42\|K04707;evm.model.utg125.45\|K08007;evm.model.utg128.1\|K05459;evm.model.utg1319.26\|K05719;evm.model.utg132.128\|K02991;evm.model.utg133.144\|K04437;evm.model.utg133.9\|K05692;evm.model.utg1335.18\|K19663;evm.model.utg134.16\|K04393;evm.model.utg134.3\|K10164;evm.model.utg13506.2\|K05762;evm.model.utg136.31\|K00444;evm.model.utg1365.22\|K06251;evm.model.utg1435.24\|K02235;evm.model.utg147.65\|K03258;evm.model.utg147.81\|K02105;evm.model.utg148.6\|K02991;evm.model.utg1536.17\|K02354;evm.model.utg154.141\|K11988;evm.model.utg155.30\|K07293;evm.model.utg156.28\|K07293;evm.model.utg156.29\|K07293;evm.model.utg158.33\|K02991;evm.model.utg158.44\|K05692;evm.model.utg1595.11\|K05697;evm.model.utg161.20\|K07293;evm.model.utg161.21\|K07293;evm.model.utg165.76\|K03258;evm.model.utg165.91\|K08550;evm.model.utg165.92\|K08550;evm.model.utg165.93\|K08550;evm.model.utg1651.30\|K04371;evm.model.utg166.11\|K06487;evm.model.utg169.88\|K05760;evm.model.utg181.8\|K03258;evm.model.utg181.9\|K03258;evm.model.utg184.176\|K01384;evm.model.utg186.30\|K05697;evm.model.utg1868.1\|K08109;evm.model.utg1868.2\|K08109;evm.model.utg190.38\|K02432;evm.model.utg195.56\|K16867;evm.model.utg195.71\|K09108;evm.model.utg197.23\|K02842;evm.model.utg199.65\|K04513;evm.model.utg2.73\|K05692;evm.model.utg20.122\|K05860;evm.model.utg20.124\|K05860;evm.model.utg201.33\|K04393;evm.model.utg209.8\|K04456;evm.model.utg212.3\|K04688;evm.model.utg220.19\|K10164;evm.model.utg221.13\|K08107;evm.model.utg226.35\|K03099;evm.model.utg236.62\|K10159;evm.model.utg242.44\|K16857;evm.model.utg243.79\|K00922;evm.model.utg246.52\|K02649;evm.model.utg2481.1\|K05763;evm.model.utg252.9\|K16865;evm.model.utg254.1\|K04392;evm.model.utg2623.4\|K00922;evm.model.utg263.36\|K04515;evm.model.utg265.9\|K12330;evm.model.utg273.2\|K07293;evm.model.utg273.3\|K07293;evm.model.utg274.21\|K05087;evm.model.utg274.25\|K05087;evm.model.utg2758.13\|K02677;evm.model.utg2758.9\|K02677;evm.model.utg2807.3\|K04437;evm.model.utg2807.4\|K04437;evm.model.utg284.59\|K02991;evm.model.utg2902.1\|K04375;evm.model.utg291.18\|K04366;evm.model.utg2955.1\|K05692;evm.model.utg304.103\|K09069;evm.model.utg306.7\|K09593;evm.model.utg324.14\|K19662;evm.model.utg324.16\|K19662;evm.model.utg324.17\|K19662;evm.model.utg326.25\|K07965;evm.model.utg326.26\|K07965;evm.model.utg326.27\|K07965;evm.model.utg326.52\|K00714;evm.model.utg33.159\|K06257;evm.model.utg33.22\|K06269;evm.model.utg331.14\|K00182;evm.model.utg332.1\|K02991;evm.model.utg332.5\|K04393;evm.model.utg346.33\|K02991;evm.model.utg349.7\|K09069;evm.model.utg354.79\|K09295;evm.model.utg357.10\|K04371;evm.model.utg359.33\|K12461;evm.model.utg359.42\|K06643;evm.model.utg3690.3\|K08845;evm.model.utg3690.4\|K08845;evm.model.utg3852.3\|K03258;evm.model.utg389.8\|K05692;evm.model.utg39.42\|K05725;evm.model.utg391.48\|K04513;evm.model.utg3925.6\|K07829;evm.model.utg404.13\|K05692;evm.model.utg407.90\|K00444;evm.model.utg414.35\|K12959;evm.model.utg414.54\|K04958;evm.model.utg417.16\|K04409;evm.model.utg422.5\|K01064;evm.model.utg422.6\|K00312;evm.model.utg422.65\|K12823;evm.model.utg422.7\|K00312;evm.model.utg422.8\|K00312;evm.model.utg4252.3\|K02991;evm.model.utg428.22\|K02329;evm.model.utg433.23\|K02991;evm.model.utg438.15\|K07831;evm.model.utg438.16\|K07831;evm.model.utg441.104\|K06269;evm.model.utg45.45\|K05425;evm.model.utg46.117\|K04515;evm.model.utg461.131\|K03209;evm.model.utg462.118\|K05859;evm.model.utg47.93\|K04515;evm.model.utg479.19\|K05742;evm.model.utg479.21\|K05692;evm.model.utg490.26\|K13375;evm.model.utg495.117\|K01116;evm.model.utg495.71\|K05704;evm.model.utg516.20\|K10380;evm.model.utg525.7\|K00922;evm.model.utg527.11\|K04361;evm.model.utg527.2\|K04393;evm.model.utg5339.1\|K02842;evm.model.utg546.1\|K10164;evm.model.utg56.147\|K02375;evm.model.utg56.70\|K02432;evm.model.utg584.48\|K06497;evm.model.utg590.62\|K16338;evm.model.utg590.87\|K01403;evm.model.utg5916.1\|K08109;evm.model.utg597.53\|K16336;evm.model.utg598.12\|K07830;evm.model.utg62.13\|K04392;evm.model.utg642.11\|K06625;evm.model.utg642.25\|K04441;evm.model.utg643.59\|K02649;evm.model.utg643.60\|K02649;evm.model.utg6589.2\|K02991;evm.model.utg66.2\|K07827;evm.model.utg660.12\|K07293;evm.model.utg661.10\|K06106;evm.model.utg661.27\|K04503;evm.model.utg661.73\|K04688;evm.model.utg661.76\|K06269;evm.model.utg67.84\|K04345;evm.model.utg672.6\|K04393;evm.model.utg688.18\|K04362;evm.model.utg6917.3\|K10164;evm.model.utg716.4\|K01365;evm.model.utg723.39\|K00714;evm.model.utg76.128\|K05085;evm.model.utg76.130\|K05085;evm.model.utg76.131\|K05085;evm.model.utg76.5\|K10164;evm.model.utg76.56\|K01357;evm.model.utg76.57\|K00445;evm.model.utg762.6\|K05692;evm.model.utg772.118\|K17457;evm.model.utg78.27\|K01398;evm.model.utg78.28\|K01398;evm.model.utg8.93\|K02187;evm.model.utg84.108\|K03156;evm.model.utg865.29\|K06225;evm.model.utg9.31\|K10380;evm.model.utg900.1\|K04959;evm.model.utg900.3\|K04959;evm.model.utg91.40\|K03258;evm.model.utg915.31\|K04366;evm.model.utg915.45\|K05692;evm.model.utg92.8\|K13376;evm.model.utg921.41\|K04692;evm.model.utg94.33\|K05083;evm.model.utg95.9\|K04456;evm.model.utg97.129\|K04660;evm.model.utg97.130\|K04660;evm.model.utg97.15\|K02991;evm.model.utg97.55\|K06270 |
| Calcium signaling pathway | map04020 | 7 | 213 | 68 | 7320 | 0.005756 | 0.194565 | evm.model.utg10.28\|K05862;evm.model.utg10.58\|K05863;evm.model.utg1012.6\|K13241;evm.model.utg1034.18\|K04961;evm.model.utg1070.30\|K04718;evm.model.utg11.242\|K04227;evm.model.utg11.243\|K04227;evm.model.utg11.293\|K05850;evm.model.utg11162.2\|K05863;evm.model.utg113.118\|K00907;evm.model.utg113.119\|K00907;evm.model.utg113.120\|K00907;evm.model.utg113.121\|K00907;evm.model.utg1139.26\|K15040;evm.model.utg114.22\|K04363;evm.model.utg1186.47\|K15040;evm.model.utg120.57\|K04198;evm.model.utg12055.1\|K07190;evm.model.utg123.31\|K01242;evm.model.utg1239.10\|K04258;evm.model.utg1239.37\|K04344;evm.model.utg1335.18\|K19663;evm.model.utg136.10\|K04851;evm.model.utg1365.2\|K13241;evm.model.utg1365.6\|K13241;evm.model.utg137.1\|K05863;evm.model.utg1385.3\|K04637;evm.model.utg1385.4\|K04635;evm.model.utg140.76\|K05863;evm.model.utg143.4\|K15041;evm.model.utg1435.11\|K05857;evm.model.utg145.127\|K00907;evm.model.utg147.136\|K05857;evm.model.utg154.124\|K04161;evm.model.utg154.52\|K13242;evm.model.utg1549.19\|K04129;evm.model.utg158.4\|K04260;evm.model.utg158.56\|K04262;evm.model.utg1585.22\|K00911;evm.model.utg1585.24\|K00911;evm.model.utg1595.50\|K02183;evm.model.utg160.72\|K04323;evm.model.utg1622.23\|K04222;evm.model.utg1622.24\|K04222;evm.model.utg1622.26\|K04222;evm.model.utg165.5\|K04603;evm.model.utg165.6\|K04603;evm.model.utg165.9\|K04603;evm.model.utg1651.10\|K05853;evm.model.utg1651.72\|K00871;evm.model.utg1651.84\|K16058;evm.model.utg169.51\|K16056;evm.model.utg169.61\|K05220;evm.model.utg1776.4\|K00911;evm.model.utg1776.5\|K00911;evm.model.utg194.37\|K04166;evm.model.utg195.74\|K08049;evm.model.utg197.67\|K05216;evm.model.utg198.57\|K04211;evm.model.utg2.16\|K04248;evm.model.utg20.122\|K05860;evm.model.utg20.124\|K05860;evm.model.utg20.73\|K04163;evm.model.utg200.64\|K04141;evm.model.utg203.135\|K08042;evm.model.utg203.136\|K08042;evm.model.utg203.140\|K08042;evm.model.utg21.21\|K04224;evm.model.utg21.54\|K04348;evm.model.utg215.22\|K05863;evm.model.utg222.115\|K04137;evm.model.utg2275.3\|K13241;evm.model.utg2401.1\|K05863;evm.model.utg242.31\|K05858;evm.model.utg25.18\|K05863;evm.model.utg2597.3\|K13241;evm.model.utg263.36\|K04515;evm.model.utg263.39\|K05089;evm.model.utg263.61\|K04142;evm.model.utg2758.13\|K02677;evm.model.utg2758.9\|K02677;evm.model.utg276.1\|K08044;evm.model.utg276.3\|K04297;evm.model.utg283.37\|K06268;evm.model.utg2891.19\|K05221;evm.model.utg295.7\|K04267;evm.model.utg296.25\|K05863;evm.model.utg31.11\|K05863;evm.model.utg319.7\|K02183;evm.model.utg319.9\|K02183;evm.model.utg324.14\|K19662;evm.model.utg324.16\|K19662;evm.model.utg324.17\|K19662;evm.model.utg3241.1\|K04157;evm.model.utg329.12\|K04130;evm.model.utg33.150\|K08043;evm.model.utg33.151\|K08043;evm.model.utg338.54\|K05863;evm.model.utg358.17\|K04633;evm.model.utg359.110\|K04226;evm.model.utg367.41\|K05858;evm.model.utg371.12\|K05862;evm.model.utg375.32\|K05862;evm.model.utg383.59\|K05211;evm.model.utg384.80\|K04157;evm.model.utg39.131\|K08048;evm.model.utg39.132\|K08048;evm.model.utg39.134\|K08048;evm.model.utg39.135\|K08048;evm.model.utg3947.6\|K05862;evm.model.utg397.40\|K04635;evm.model.utg397.41\|K04635;evm.model.utg407.69\|K04850;evm.model.utg407.72\|K04850;evm.model.utg407.73\|K04850;evm.model.utg407.82\|K04850;evm.model.utg414.33\|K04229;evm.model.utg414.34\|K04229;evm.model.utg414.54\|K04958;evm.model.utg421.11\|K05209;evm.model.utg421.7\|K05209;evm.model.utg421.8\|K05209;evm.model.utg423.31\|K04632;evm.model.utg423.35\|K04632;evm.model.utg427.32\|K04197;evm.model.utg427.34\|K04197;evm.model.utg428.42\|K05871;evm.model.utg433.27\|K04163;evm.model.utg435.38\|K04718;evm.model.utg441.94\|K05853;evm.model.utg443.2\|K05862;evm.model.utg45.49\|K04136;evm.model.utg451.24\|K02183;evm.model.utg46.117\|K04515;evm.model.utg46.203\|K04223;evm.model.utg46.204\|K04223;evm.model.utg46.44\|K09565;evm.model.utg462.118\|K05859;evm.model.utg467.37\|K13755;evm.model.utg468.21\|K05219;evm.model.utg47.70\|K08041;evm.model.utg47.71\|K08041;evm.model.utg47.72\|K08041;evm.model.utg47.93\|K04515;evm.model.utg479.44\|K04279;evm.model.utg490.8\|K00911;evm.model.utg495.117\|K01116;evm.model.utg513.15\|K05862;evm.model.utg513.23\|K04195;evm.model.utg516.4\|K15041;evm.model.utg5227.1\|K05863;evm.model.utg524.46\|K05862;evm.model.utg524.60\|K04852;evm.model.utg524.61\|K04852;evm.model.utg524.62\|K04852;evm.model.utg524.64\|K04852;evm.model.utg527.11\|K04361;evm.model.utg530.7\|K05863;evm.model.utg538.2\|K04161;evm.model.utg568.11\|K05849;evm.model.utg568.8\|K05849;evm.model.utg581.3\|K04194;evm.model.utg590.76\|K12042;evm.model.utg599.91\|K04854;evm.model.utg61.11\|K04604;evm.model.utg61.12\|K04604;evm.model.utg61.8\|K04604;evm.model.utg61.9\|K04604;evm.model.utg6210.2\|K07190;evm.model.utg623.22\|K13755;evm.model.utg623.23\|K13755;evm.model.utg623.24\|K13755;evm.model.utg623.26\|K13755;evm.model.utg623.29\|K13755;evm.model.utg623.31\|K13755;evm.model.utg623.34\|K05863;evm.model.utg63.75\|K05217;evm.model.utg641.52\|K04144;evm.model.utg646.32\|K04162;evm.model.utg657.1\|K04266;evm.model.utg67.168\|K04634;evm.model.utg67.169\|K04634;evm.model.utg67.170\|K04634;evm.model.utg67.171\|K04634;evm.model.utg67.84\|K04345;evm.model.utg672.11\|K00871;evm.model.utg679.12\|K05840;evm.model.utg693.3\|K05863;evm.model.utg712.26\|K16057;evm.model.utg750.46\|K04131;evm.model.utg76.128\|K05085;evm.model.utg76.130\|K05085;evm.model.utg76.131\|K05085;evm.model.utg76.66\|K05857;evm.model.utg768.24\|K04282;evm.model.utg768.25\|K04282;evm.model.utg797.13\|K05863;evm.model.utg797.61\|K04856;evm.model.utg797.62\|K04856;evm.model.utg802.4\|K05862;evm.model.utg809.2\|K13241;evm.model.utg833.26\|K04634;evm.model.utg833.27\|K04634;evm.model.utg889.2\|K05850;evm.model.utg892.51\|K05208;evm.model.utg892.52\|K05208;evm.model.utg892.80\|K04849;evm.model.utg893.2\|K04169;evm.model.utg90.15\|K05862;evm.model.utg900.1\|K04959;evm.model.utg900.3\|K04959;evm.model.utg91.41\|K07190;evm.model.utg91.53\|K00907;evm.model.utg94.33\|K05083;evm.model.utg944.15\|K05869;evm.model.utg967.64\|K04855;evm.model.utg983.10\|K04809;evm.model.utg983.31\|K04963;evm.model.utg983.35\|K04963;evm.model.utg994.3\|K04133 |
