# Supplementary material for: The reference genome and transcriptome of the limestone langur, Trachypithecus leucocephalus, reveal expansion of genes related to alkali tolerance
Source: BMC Biol. 2021 Apr 8;19:67. doi: 10.1186/s12915-021-00998-2 (PMC8034193; doi:10.1186/s12915-021-00998-2)
Supplement: Supplementary file 22 — Additional file 22: Table S17. The expression of all genes in mineral absorption pathways from seven tissues of T. leucocephalus. [file 12915_2021_998_MOESM22_ESM.docx]

| **Additional file 22 Table S17 : The expression of all genes in mineral absorption pathways from seven tissues of T. leucocephalus.** | | | | | | | | | | | | | |
| --- | --- | --- | --- | --- | --- | --- | --- | --- | --- | --- | --- | --- | --- |
| Genes in special gene family | Gene symbol | Blood | lung_1 | lung_2 | live_2 | Testis_1 | Testis_2YG | Muscle_Breast | Muscle_leg | kidney_1 | kidney_2 | Heart_1 | Heart_2 |
| evm.model.utg263.5 | ATOX1 | 339.9938 | 553.9085 | 406.0314 | 1171.379 | 176.2879 | 297.6671 | 503.9372 | 410.114 | 932.5358 | 1547.013 | 1208.424 | 3324.874 |
| evm.model.utg1077.7 | ATP7A | 8.416563 | 3.380524 | 5.876224 | 0 | 11.00823 | 14.69425 | 1.297295 | 3.684618 | 15.86861 | 18.12906 | 15.5099 | 9.015668 |
| evm.model.utg377.38 | CLCN2-1 | 0.713327 | 2.180579 | 0.87213 | 2.254159 | 3.11814 | 10.53737 | 1.650535 | 4.972015 | 1.966507 | 5.242137 | 17.93916 | 6.604248 |
| evm.model.utg377.39 | CLCN2-2 | 0.073904 | 0.198651 | 0.21187 | 0 | 0.343244 | 0 | 0 | 0 | 0.962099 | 0 | 0 | 0 |
| evm.model.utg274.91 | CYBRD | 39.69074 | 64.20532 | 200.9488 | 10.50359 | 2.118877 | 25.34216 | 6.292565 | 167.1395 | 65.92435 | 97.70608 | 0 | 17.0064 |
| evm.model.utg1033.7 | FTH1-1 | 18.98004 | 7.521865 | 15.95757 | 19.60823 | 26.55866 | 16.75529 | 3.480604 | 4.119049 | 14.5718 | 30.39982 | 13.00393 | 13.10225 |
| evm.model.utg1513.5 | FTH1-18 | 0 | 0 | 1.012908 | 0 | 0 | 0 | 0 | 0 | 0 | 0 | 0 | 0 |
| evm.model.utg160.12 | FTH1-16 | 0 | 0 | 0.236317 | 0 | 0.255232 | 0 | 0 | 0 | 2.14622 | 0 | 0 | 15.02256 |
| evm.model.utg212.28 | FTH1-3 | 104.6329 | 173.2547 | 124.0451 | 105.7495 | 22.73926 | 62.55916 | 24.36659 | 71.76996 | 104.0823 | 113.5037 | 86.31597 | 81.533 |
| evm.model.utg229.40 | FTH1-10 | 0 | 0 | 0 | 0 | 6.486357 | 11.87914 | 0 | 0 | 0 | 0 | 0 | 0 |
| evm.model.utg256.5 | FTH1-11 | 0 | 0 | 1.089205 | 0 | 0 | 6.155553 | 0 | 0 | 0.659475 | 0 | 0 | 0 |
| evm.model.utg288.82 | FTH1-12 | 0 | 1.273667 | 0 | 0 | 15.84524 | 6.909295 | 0 | 0 | 0 | 0 | 0 | 0 |
| evm.model.utg306.32 | FTH1-7 | 2.6451 | 5.055974 | 3.665152 | 18.94635 | 2.184014 | 2.857008 | 5.675256 | 2.985007 | 3.673025 | 0 | 18.84747 | 2.921533 |
| evm.model.utg326.37 | FTH1-9 | 0.3045 | 1.636975 | 0.763832 | 0 | 0.707119 | 1.233353 | 0 | 0 | 1.189217 | 6.340217 | 0 | 2.522416 |
| evm.model.utg329.43 | FTH1-5 | 8.328233 | 19.12992 | 17.36414 | 56.94202 | 4.922933 | 7.359901 | 5.414798 | 7.689638 | 15.3758 | 31.5288 | 53.94748 | 16.3066 |
| evm.model.utg396.48 | FTH1-6 | 12.95194 | 11.73977 | 7.771625 | 8.090604 | 2.098424 | 8.540137 | 6.462634 | 0 | 7.058164 | 6.271674 | 5.365587 | 1.247574 |
| evm.model.utg4070.5 | FTH1-8 | 17.52592 | 4.026431 | 3.059729 | 0 | 1.391428 | 0 | 0 | 0 | 7.605235 | 0 | 24.01533 | 0 |
| evm.model.utg416.11 | FTH1-13 | 0.288723 | 0.776079 | 1.138116 | 0 | 0.893975 | 0 | 2.064918 | 0 | 0 | 0 | 0 | 0 |
| evm.model.utg4989.3 | FTH1-14 | 0.151422 | 2.035098 | 1.193785 | 16.26915 | 0 | 0 | 0.54148 | 0 | 0 | 0 | 0 | 2.508708 |
| evm.model.utg506.22 | FTH1-4 | 91.1563 | 193.7413 | 139.1302 | 113.884 | 32.3507 | 153.3313 | 30.86435 | 171.7353 | 115.1214 | 201.7843 | 107.895 | 75.26123 |
| evm.model.utg568.67 | FTH1-19 | 0.949832 | 0 | 0 | 0 | 0 | 0 | 0 | 0 | 0 | 0 | 0 | 0 |
| evm.model.utg63.169 | FTH1-2 | 10247.59 | 21403.32 | 18993.1 | 12196.75 | 2190.223 | 4096.241 | 3960.589 | 8933.707 | 16743.47 | 19628.53 | 11870.59 | 10157.16 |
| evm.model.utg866.14 | FTH1-17 | 0 | 0 | 0 | 0 | 1.782408 | 1.865319 | 0 | 0 | 0 | 0 | 0 | 0 |
| evm.model.utg875.18 | FTH1-15 | 0.369029 | 0.330647 | 1.322434 | 0 | 0 | 0 | 0 | 0 | 1.441237 | 0 | 0 | 0 |
| evm.model.utg9008.1 | FTH1-20 | 0 | 0 | 0 | 0 | 0 | 1.85003 | 0 | 0 | 0 | 0 | 0 | 0 |
| evm.model.utg42.36 | FTL | 0 | 0.651231 | 0 | 0 | 0 | 0 | 0 | 0 | 0 | 0 | 0 | 0 |
| evm.model.utg83.3 | FTL | 0 | 0.853466 | 0 | 0 | 48.29564 | 7.716363 | 0 | 0 | 1.240038 | 0 | 0 | 0 |
| evm.model.utg1379.1 | HMOX1 | 44.52228 | 25.30122 | 9.98438 | 50.56628 | 12.09508 | 6.100098 | 8.078293 | 15.93348 | 145.8197 | 86.23552 | 3.353492 | 21.83254 |
| evm.model.utg11.412 | HMOX2-1 | 5.224073 | 0.222892 | 0.178292 | 0 | 6.162039 | 0 | 0 | 0 | 1.943096 | 0 | 0 | 0 |
| evm.model.utg11.413 | HMOX2-2 | 4.985782 | 0.788333 | 1.050987 | 0 | 5.44854 | 1.187914 | 3.146282 | 0 | 0.381801 | 6.10663 | 0 | 0 |
| evm.model.utg195.63 | HMOX2-3 | 262.1815 | 69.14283 | 72.1899 | 151.0927 | 824.3136 | 323.2475 | 29.54396 | 26.78032 | 100.4607 | 84.18288 | 78.28341 | 66.25521 |
| evm.model.utg461.85 | SLC11 | 8.537019 | 13.83791 | 27.36666 | 0 | 1.922419 | 6.91571 | 0 | 2.627472 | 12.32612 | 3.231921 | 0 | 6.429 |
| evm.model.utg155.26 | SLC26-1 | 21.87343 | 22.13656 | 2.80128 | 0 | 3.053263 | 3.195289 | 0 | 0.60699 | 5.601717 | 0 | 5.110083 | 0 |
| evm.model.utg320.50 | SLC26-2 | 0 | 1.313888 | 2.218751 | 0 | 1.38736 | 0 | 0 | 0 | 1.166615 | 0 | 0 | 0 |
| evm.model.utg154.83 | SLC30-1 | 0 | 0 | 0 | 0 | 108.058 | 109.3616 | 0 | 0 | 0 | 0 | 0 | 0 |
| evm.model.utg58.69 | SLC30-2 | 7.54152 | 6.194043 | 4.604326 | 11.25385 | 21.62119 | 19.23289 | 0.249705 | 0 | 7.454215 | 8.723757 | 12.43902 | 10.41208 |
| evm.model.utg183.40 | SLC31 | 17.41358 | 11.00381 | 21.80799 | 0 | 4.54045 | 5.939569 | 2.621902 | 0 | 57.27019 | 122.1326 | 13.06097 | 13.66585 |
| evm.model.utg600.62 | SLC39 | 3.743255 | 6.441059 | 2.103579 | 0 | 1.778054 | 1.378343 | 0 | 0 | 0.443006 | 0 | 0 | 28.54182 |
| evm.model.utg1403.2 | SLC5A-1 | 0 | 0 | 0 | 0 | 4.069271 | 8.517118 | 0 | 0 | 1.368722 | 7.297231 | 0 | 0 |
| evm.model.utg515.13 | SLC5A-2 | 0 | 0.443146 | 0 | 0 | 0 | 0 | 0 | 0 | 7.726392 | 0 | 0 | 0 |
| evm.model.utg641.15 | SLC5A-3 | 412.0071 | 451.6779 | 286.7017 | 248.1678 | 172.3136 | 182.434 | 51.62294 | 100.1911 | 742.7123 | 414.8079 | 133.7227 | 1152.81 |
| evm.model.utg203.25 | SLC6A | 0 | 0 | 0 | 0 | 17.83165 | 22.12781 | 0 | 0 | 20.74329 | 28.43774 | 0 | 0 |
| evm.model.utg203.9 | SLC9A | 10.79242 | 0.143471 | 0.137716 | 0 | 1.388229 | 1.816006 | 0 | 0 | 6.003496 | 1.333632 | 0 | 0 |
| evm.model.utg190.24 | STEAP | 0 | 2.877679 | 0.92075 | 0 | 0 | 0.650443 | 0 | 0 | 3.344887 | 0 | 0 | 1.330266 |
| evm.model.utg67.127 | TRPM6-1 | 0.01855 | 0.698058 | 0.146243 | 0 | 2.096406 | 0.751344 | 0 | 0 | 1.49721 | 0 | 0 | 0.61465 |
| evm.model.utg67.128 | TRPM6-2 | 14.07448 | 5.14719 | 3.602611 | 7.715267 | 29.79378 | 65.15156 | 1.027137 | 0 | 7.852511 | 5.98072 | 5.116668 | 2.379393 |
| evm.model.utg67.130 | TRPM6-3 | 13.97245 | 29.65857 | 21.22053 | 22.33973 | 22.91911 | 24.25472 | 6.543015 | 11.26283 | 64.09702 | 45.025 | 2.963086 | 99.21002 |
| evm.model.utg578.42 | TRPV6-4 | 0 | 0.206313 | 0.082516 | 0 | 0.356482 | 0 | 0 | 0 | 0.799364 | 0 | 0 | 0 |
| evm.model.utg461.16 | VDR | 14.4157 | 433.711 | 375.005 | 55.56424 | 25.32012 | 20.4233 | 2.773985 | 4.377087 | 66.1464 | 72.68448 | 16.12166 | 228.1236 |
