# Supplementary material for: The reference genome and transcriptome of the limestone langur, Trachypithecus leucocephalus, reveal expansion of genes related to alkali tolerance
Source: BMC Biol. 2021 Apr 8;19:67. doi: 10.1186/s12915-021-00998-2 (PMC8034193; doi:10.1186/s12915-021-00998-2)
Supplement: Supplementary file 23 — Additional file 23: Table S18. Annotation analysis of positive selected genes related to alkaline ion metabolism of T. leucocephalus. [file 12915_2021_998_MOESM23_ESM.docx]

| **Additional file 23: Table S18: Annotation analysis of positive selected genes related to alkaline ion metabolism of T. leucocephalus.** | | | | | | | | | | | | |
| --- | --- | --- | --- | --- | --- | --- | --- | --- | --- | --- | --- | --- |
| Species | site 10 | site 85 | site 87 | site 92 | site 99 | site 117 | site 127 | site 136 | site 142 | site 146 | site 151 | site 152 |
| P. paniscus | A | E | K | P | P | Q | T | V | D | R | K | V |
| T. leucocephalus | G | G | A | A | A | G | A | I | I | I | G | I |
| C. sabaeus | A | E | K | P | P | Q | T | V | D | R | K | V |
| M. mulatta | A | E | K | P | P | Q | T | V | D | R | K | V |
| H. sapiens | A | E | K | P | P | Q | T | V | D | R | K | V |
| R. roxellana | A | E | K | P | P | Q | T | V | D | K | K | V |
| M. fascicularis | A | E | K | P | P | Q | T | V | D | R | K | V |
| P. tephrosceles | A | E | K | P | P | Q | T | V | D | R | K | V |
| P. anubis | A | E | K | P | P | Q | T | V | D | R | K | V |
| C. angolensis | A | E | K | P | P | Q | T | V | D | R | K | V |
| M. nemestrina | A | E | K | P | P | Q | T | V | D | R | K | V |
| R. bieti | A | E | K | P | P | Q | T | V | D | R | K | V |
| P. troglodytes | A | E | K | P | P | Q | T | V | D | R | K | V |
| N. leucogenys | A | E | K | P | P | Q | T | V | D | R | K | V |
| C. atys | A | E | K | P | P | Q | T | V | D | R | K | V |
